# Differential sensitivity to hypoxia enables shape‐based classification of sickle cell disease and trait blood samples at point of care

**DOI:** 10.1002/btm2.10643

**Published:** 2023-12-27

**Authors:** Claudy D'Costa, Oshin Sharma, Riddha Manna, Minakshi Singh, Samrat Singh, Srushti Singh, Anish Mahto, Pratiksha Govil, Sampath Satti, Ninad Mehendale, Yazdi Italia, Debjani Paul

**Affiliations:** ^1^ Department of Biosciences and Bioengineering Indian Institute of Technology Bombay Mumbai India; ^2^ MedPrime Technologies Pvt. Ltd. Casa Piedade Co‐operative Housing Society Thane India; ^3^ Shirin and Jamshed Guzder Regional Blood Centre Valsad India; ^4^ Wadhwani Research Centre for Bioengineering Indian Institute of Technology Bombay Mumbai India

**Keywords:** diagnostics, hemoglobin polymerization, RBC shape, shape‐based classifier, sickle cell anemia, smartphone microscopy

## Abstract

Red blood cells (RBCs) become sickle‐shaped and stiff under hypoxia as a consequence of hemoglobin (Hb) polymerization in sickle cell anemia. Distinguishing between sickle cell disease and trait is crucial during the diagnosis of sickle cell disease. While genetic analysis or high‐performance liquid chromatography (HPLC) can accurately differentiate between these two genotypes, these tests are unsuitable for field use. Here, we report a novel microscopy‐based diagnostic test called ShapeDx™ to distinguish between disease and trait blood in less than 1 h. This is achieved by mixing an unknown blood sample with low and high concentrations of a chemical oxygen scavenger and thereby subjecting the blood to slow and fast hypoxia, respectively. The different rates of Hb polymerization resulting from slow and fast hypoxia lead to two distinct RBC shape distributions in the same blood sample, which allows us to identify it as healthy, trait, or disease. The controlled hypoxic environment necessary for differential Hb polymerization is generated using an imaging microchamber, which also reduces the sickling time of trait blood from several hours to just 30 min. In a single‐blinded proof‐of‐concept study conducted on a small cohort of clinical samples, the results of the ShapeDx™ test were 100% concordant with HPLC results. Additionally, our field studies have demonstrated that ShapeDx™ is the first reported microscopy test capable of distinguishing between sickle cell disease and trait samples in resource‐limited settings with the same accuracy as a gold standard test.

**Translational Impact Statement**

We report a point‐of‐care microscopy test to distinguish between individuals with sickle cell anemia and its carriers. We subject an unknown blood sample to both slow and fast deoxygenation for 30 min. As a result, the RBCs deform into characteristic shapes. By comparing the two images of deformed RBCs under slow and fast deoxygenation against an image‐based classifier, we can differentiate carriers from anemic individuals. Fast detection, portability, and accuracy of this test make it ideal for early detection and subsequent management of sickle cell disease.

## 1 INTRODUCTION

Sickle cell anemia is a genetic disorder caused by a glutamine to valine mutation in the β‐globin gene.^1^ This mutation results in complete or partial replacement of normal adult hemoglobin (HbA) by sickle hemoglobin (HbS). Complete replacement of HbA leads to sickle cell disease (homozygous; SCD), while partial replacement leads to sickle cell trait (heterozygous; SCT). Often sickle cell trait is co‐manifested with other hemoglobinopathies such as beta‐thalassemia.^2^ The sickle gene responsible for these sub‐types is widely prevalent in several parts of the world including sub‐Saharan Africa, Latin America, Middle East, and India.^3^ More than 500 children die of sickle cell anemia every day in low‐ and medium‐income countries (LMIC) due to a lack of effective newborn screening programs.^4^, ^5^

A robust public health strategy to manage sickle cell anemia needs to distinguish between SCD and SCT genotypes such that resources for clinical and non‐clinical interventions can be appropriately allocated.^6^ Unlike HbA, HbS polymerizes under hypoxic conditions. As a result, red blood cells (RBCs) containing HbS become rigid and sickle‐shaped.^1^ Since SCD blood contains 80%–95% HbS and no HbA, the stiffening of RBCs leads to frequent vaso‐occlusive crisis (also known as sickle cell crisis), joint pain, spleen damage, increased susceptibility to infection and life‐threatening anemia.^1^ Early interventions such as penicillin prophylaxis, hydroxyurea, etc. can provide relief and reduce the mortality rates in SCD by as much as 70%.^7^ In contrast, SCT is manifested as a relatively benign condition as RBCs in SCT contain 55%–65% HbA and 30%–40% HbS.^8^ However, there is an increased risk of exertional injury, renal abnormalities, venous thromboembolism, etc. in SCT.^9^

SCD and SCT are differentiated using gold standard tests such as high‐performance liquid chromatography (HPLC) or isoelectric focusing (IEF).^10^ Although these tests exhibit high sensitivity and specificity, recent blood transfusion or sample degradation during transport can potentially affect their accuracy.^3^ Moreover, unavailability of laboratory infrastructure and a shortage of trained personnel in the endemic areas of LMICs hinder the widespread implementation of these tests. It takes several days for HPLC results from centralized laboratories to reach patients in the endemic areas.^6^ Consequently, these gold‐standard tests often prove inadequate for sustaining large‐scale mass screening programs in LMICs.^11^ There is a pressing need for a simple confirmatory test that can be conducted by minimally trained personnel at the screening site itself, providing results within an hour.

Several excellent point‐of‐care (POC) tests have been reported recently^7^, ^12^, ^13^, ^14^, ^15^ for distinguishing between SCD and SCT. While SickleSCAN^16^ and HemoTypeSC^17^ are immunoassays that give results in 5–10 min, these are qualitative tests. While such tests are ideal for screening in the remote locations, the results need confirmation by additional diagnostic tests. HemeChip is a cellulose acetate electrophoresis test that identifies various hemoglobin types (C, A2, S, F, A0) from venous blood within 15 min.^18^ It shows high diagnostic accuracy when the electrophoresis chip is imaged with the help of a Gazelle reader.^18^ SickleCERT is an absorption‐based test and needs a customized spectrophotometer.^19^ Table S1 shows a comparison between the existing POC tests. Due to the magnitude of the population that needs screening in affected LMICs,^4^, ^5^ we need to supplement existing tests with other POC technologies.

Despite the initial use of microscopy to study the shape of sickle RBCs,^3^, ^5^, ^10^, ^20^ it lacks the ability to differentiate between SCD and SCT blood samples due to the absence of a clear correlation between the normoxic morphology of RBCs and sickle blood genotypes. Recent advancements in microscopy and deep learning techniques led to numerous experimental and computational studies focused on classifying the diverse RBC shapes present in sickle blood.^12^, ^21^, ^22^, ^23^, ^24^, ^25^, ^26^, ^27^, ^28^ For example, Van Beers et al. utilized imaging flow cytometry on SCD samples to identify sickle RBCs based on a shape ratio.^21^ Jung et al. employed quantitative phase imaging to examine a mixture of RBCs from SCD and SCT samples, analyzing aspects such as aspect ratio, Hb content, membrane curvature, and membrane fluctuation.^22^ Xu et al. utilized a deep convolutional neural network to classify the shapes of RBCs from SCD patients under oxygenation and deoxygenation.^28^ Javidi et al. identified a combination of morphological and spatio‐temporal features to differentiate between RBCs from healthy individuals and those with SCD.^23^ Praljak et al. developed a deep neural network to automate the classification of RBCs from SCD samples under shear stress.^24^ Delgado‐Font et al. developed an automated classification scheme based on the morphologies of sickle RBCs in peripheral blood smears.^25^ De Haan et al. designed a microscope attachment for smartphones to image blood smears and employed deep learning techniques to classify sickle RBCs based on their shapes.^26^ The authors also claimed that blood smear images cannot differentiate between sickle cell genotypes.^26^ Ilyas et al. developed an optical attachment for smartphones to image sickle RBCs in a microfluidic chip, aiming to diagnose SCD in resource‐limited settings.^12^ All these reports either classify different RBC shapes typically seen in sickle blood or they only distinguish between healthy and sickle blood samples. While multiple reports^27^, ^29^, ^30^, ^31^ connect the RBC shapes in SCD blood to deoxygenation rate, none extended this concept to a diagnostic technology so far. Asakura et al.^29^, ^32^ varied the deoxygenation rate to showcase the varying proportions of classically sickle and granular cells in SCD blood. Horiuchi et al.^29^ also demonstrated the same using gas exchange for deoxygenation. However, similar studies in SCT blood do not exist.

These microscopy tests do not differentiate between SCD and SCT blood due to the following two reasons. Firstly, most microscopy studies^26^, ^27^, ^28^, ^32^, ^33^ utilize RBCs from SCD blood, with limited attention given to SCT samples.^22^ SCT individuals have been overlooked due to their milder clinical manifestations compared to SCD patients. However, recent studies revealed that SCT patients face increased risks of exertional injury, renal abnormalities, and venous thromboembolism compared to healthy individuals.^9^ Hence, accurately diagnosing SCT is crucial for providing appropriate genetic counseling and making informed policy decisions. Secondly, existing microscopy reports image blood samples under either normoxic or a single hypoxic condition.^34^ We need to treat blood with *more than one* concentration of an oxygen scavenger to enable shape‐based classification of SCD and SCT blood samples.

**FIGURE 1:**
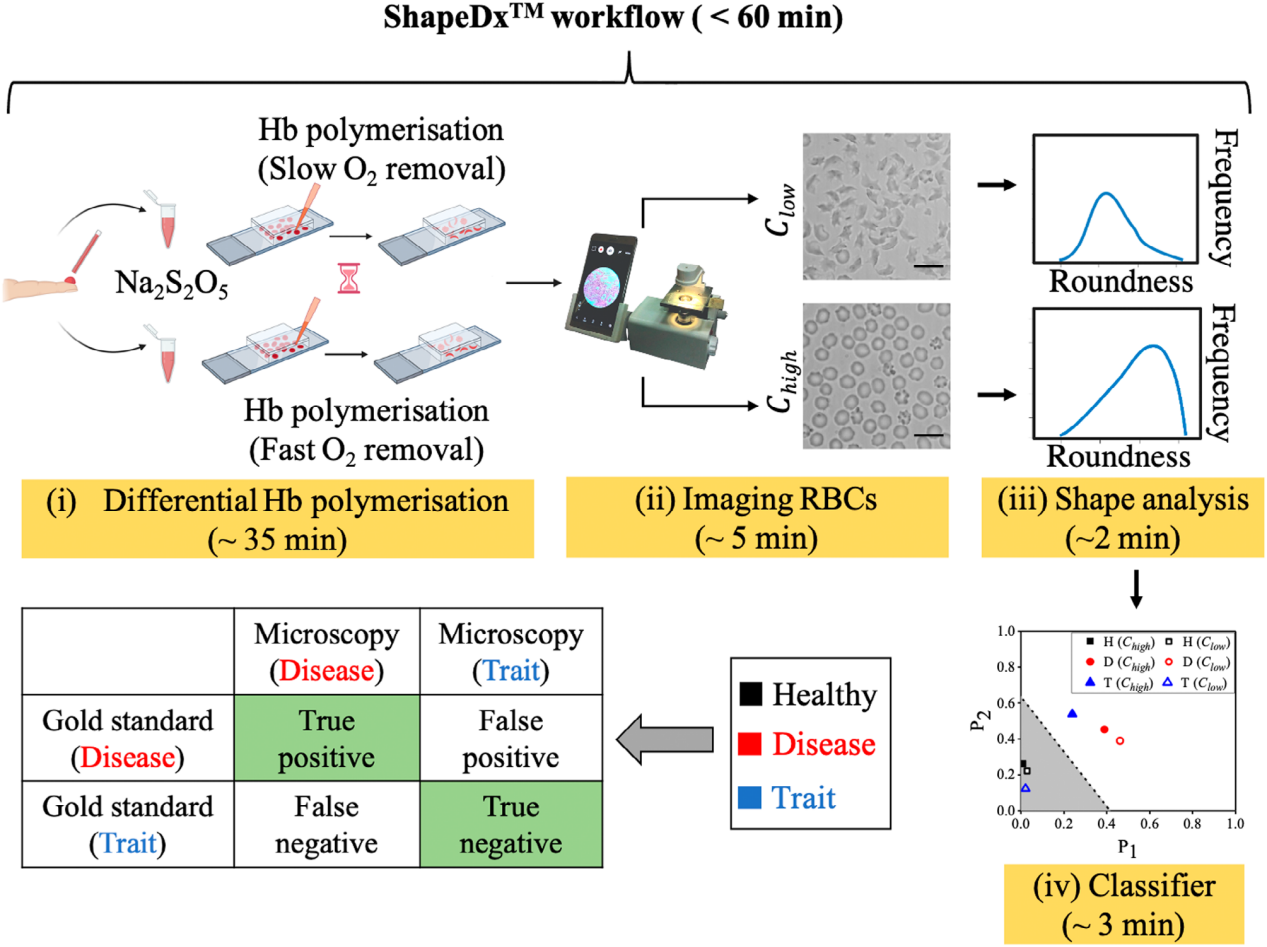
Schematic diagram of our microscopy test. The ShapeDx™ work flow involves treating each blood sample with two oxygen scavenger concentrations, imaging them with a portable smartphone microscope and comparing the RBC shape distributions in the two images to arrive at a diagnosis. Compared to a gold standard test (HPLC) that takes at least 48 h from sample to diagnosis, ShapeDx™ gives results in less than an hour. The scale bar equals 10 μm.

We report a microscopy test, ShapeDx™ (Figure 1), where an unknown blood sample is subjected to both slow and fast deoxygenation to definitively determine whether it is SCD or SCT blood. This hypothesis is supported by the fact that the shape of sickle RBCs under partial hypoxia is determined by both intrinsic (e.g., HbS content, presence of HbAS heterodimer, and the nature of polymerization) and extrinsic factors (e.g., the rate of deoxygenation).^35^, ^36^, ^37^, ^38^ When SCD blood is slowly deoxygenated (i.e., annealed), it exhibits a combination of sickle‐shaped, holly leaf, and granular RBCs^27^, ^29^, ^32^ whereas fast deoxygenation (i.e., quenching) leads to primarily granular RBCs.^32^, ^39^ The lower HbS concentration and the presence of HbAS heterodimers in SCT blood^35^, ^36^ result in fewer nucleation sites. Consequently, we hypothesize that most RBCs in SCT blood will retain their biconcave shape under slow hypoxia and will appear indistinguishable from healthy RBCs. However, under fast deoxygenation, these RBCs will deform primarily into holly leaf shapes due to heterogeneous nucleation. See Figure S1 for a discussion relating RBC shapes to hemoglobin polymerization.

The ShapeDx™ workflow (Figure 1) for identifying an unknown sample involves the following steps: (a) mixing two finger‐prick volumes of blood from each sample with two concentrations of a chemical oxygen scavenger (sodium metabisulphite; SMBS) and loading them into two imaging microchambers to facilitate slow and fast Hb polymerization, (b) imaging the RBC shapes in each chip after 30 minutes using a smartphone microscope, (c) generating shape distributions from the captured images, and finally, (d) comparing the shape distributions against a classifier to obtain a definitive diagnosis. During validation, each unknown blood sample is independently analyzed using HPLC as the gold standard test, and the results are compared. We developed and trained this shape‐based classifier using 164 known blood samples. Additionally, we conducted a proof‐of‐concept single‐blinded study with 35 unknown samples to validate this classifier, achieving 100% concordance compared to the gold standard (HPLC) test. Compared to the HPLC test, which requires at least 48 hours from sample collection to result communication to the patient and relies on a clinician's interpretation of the hemoglobin profile for diagnosis, the ShapeDx™ test delivers results within an hour from sample collection, without requiring any clinician's input.

## 2 RESULTS

### 2.1 0.1% and 0.3% SMBS led to slow and fast deoxygenation of blood

**FIGURE 2:**
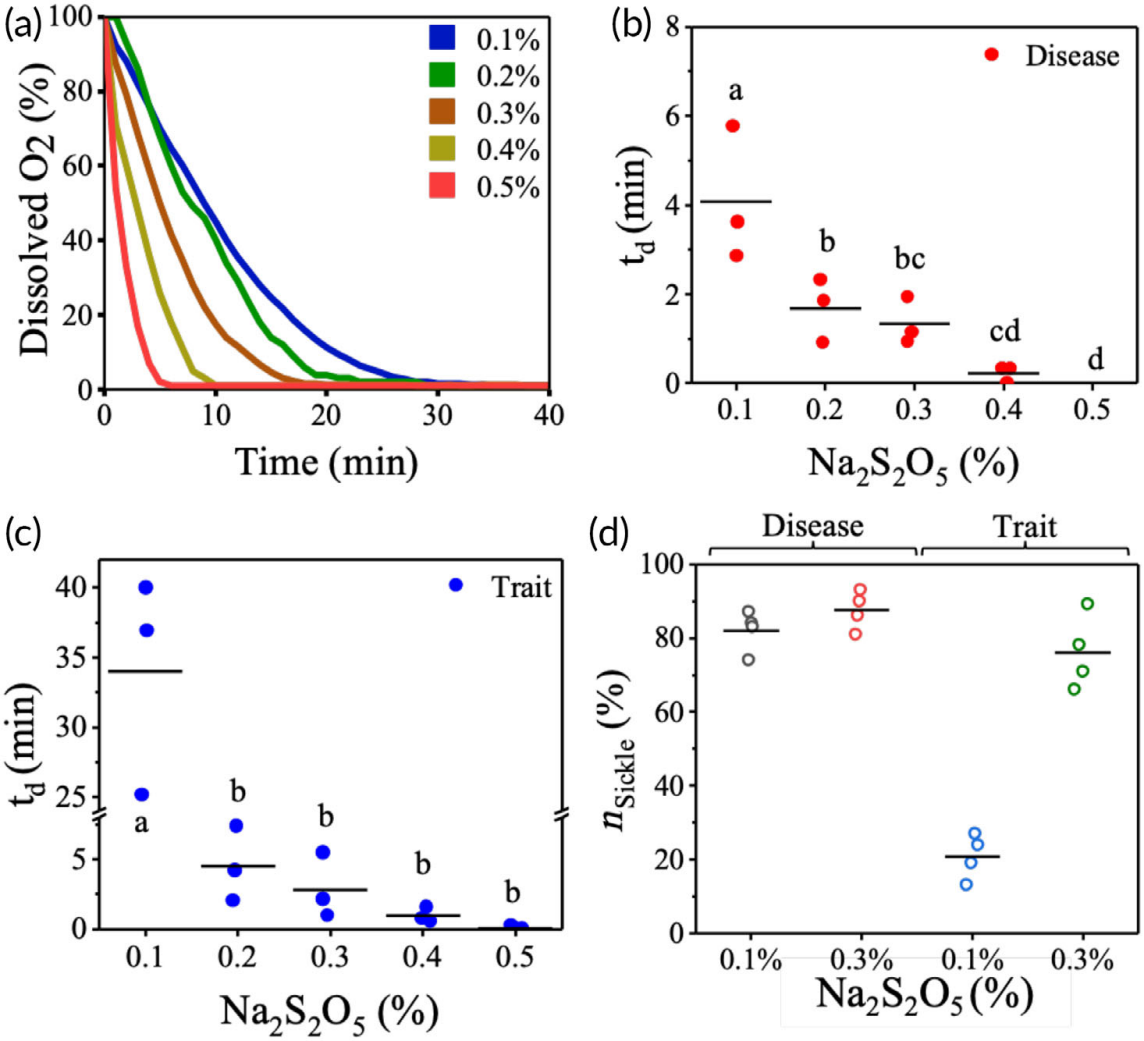
Optimization of sodium metabisulphite (SMBS) concentration to induce slow and fast polymerization in disease and trait blood samples. (a) Decrease in oxygen content with time for different concentrations of SMBS. (b) $t_{d}$ as a function of SMBS concentration in three SCD samples. (c) $t_{d}$ as a function of SMBS concentration in three SCT samples. (d) Percentage of sickled RBCs $\left(n_{\text{sickle}}\right)$ in disease (N = 4) and trait (N = 4) samples treated with 0.1% and 0.3% SMBS, respectively. The horizontal lines indicate the mean values in all the plots in b, c, and d. Different letters indicate significant difference (P = 0.05), as determined by one‐way ANOVA.

The standard sickling assays take at least 1–2 h when SCD blood is mixed with 2% SMBS.^32^ Considering that SCD and SCT samples contain varying amounts of HbS, we propose that we can achieve different levels of HbS polymerization by controlling the deoxygenation rates in these samples. To shorten the duration of the sickling assay, we employ an imaging microchamber. In order to achieve different deoxygenation rates, we conduct sickling assays using 20X diluted blood and SMBS concentrations ranging from 0.1% to 0.5%. Figure 2a illustrates a plot of the dissolved oxygen content over time for different SMBS concentrations. Each curve is fitted to the function $y={\mathrm{Ae}}^{-\frac{x}{\tau }}+B$, where y indicates the dissolved oxygen content (%), x indicates time (min), τ indicates a time constant (min) and A,B are fitting parameters. We use this function to obtain the corresponding time constants (Table S2), which ranges from 12.2 to 1.6 min. Even with a concentration of 0.1% SMBS, the oxygen content decreases to less than 5% within 30 min, representing the physiological level of deoxygenation in blood.^40^

Next, we record real time sickling videos and determine the time point (delay time “$t_{d}$” explained in Figure S2) at which the first unsickled RBC in the field of view starts sickling. We use $t_{d}$ as a parameter to compare the sickling behavior of SCD and SCT RBCs treated with different SMBS concentrations. Figure 2b,c show how $t_{d}$ varies with SMBS concentration for three SCD and three SCT samples. For SCD samples, the value of $t_{d}$ reduced from 4.1 min ± 0.9 min (mean ± SEM) to (1.3 ± 0.3) min for 0.1% and 0.3% SMBS, respectively. The corresponding time points for SCT samples are (33.4 ± 4.8) min and (2.8 ± 1.3) min. Sickling occurred almost instantaneously with 0.4% and 0.5% SMBS for both SCD and SCT samples. Figure 2d shows the percentage of sickle RBCs ($n_{\text{sickle}}$) in four SCD and four SCT samples treated with both 0.1% and 0.3% SMBS. While 82 ± 3% (mean ± SEM) of RBCs in SCD blood sickle within 30 min when treated with 0.1% SMBS, only 21 ± 3% of RBCs in SCT blood sickle at this concentration. In contrast, the numbers of sickled RBCs in SCD and SCT blood samples treated with 0.3% SMBS for 30 min are 88 ± 3% and 76 ± 5%, respectively. There is a significant difference in the mean $t_{d}$ and the mean $n_{\text{sickle}}$ values between SCD and SCT samples at 0.1% SMBS concentration. Therefore, 0.1% was chosen as the “low” oxygen scavenger concentration ($C_{\mathrm{low}}$) resulting in slow hemoglobin polymerization. We chose 0.3% as the “high” oxygen scavenger concentration ($C_{\text{high}}$) for fast HbS polymerization in subsequent experiments.

### 2.2 Treating sickle blood with $C_{\mathrm{low}}$ and $C_{\text{high}}$ generates characteristic populations of sickle, holly leaf and granular RBCs

**FIGURE 3:**
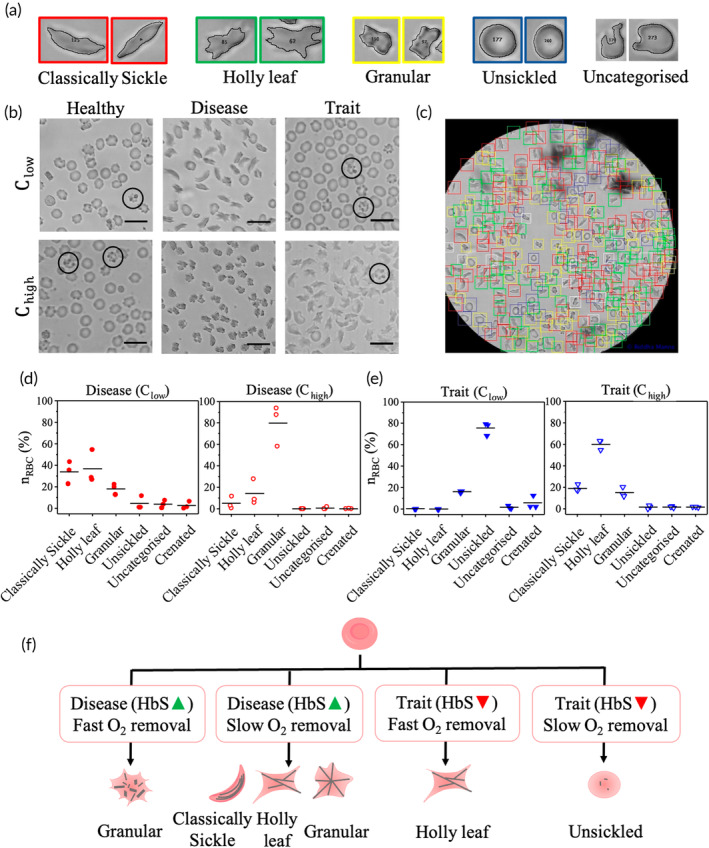
(a) Typical RBC shapes seen in SCD blood: classically sickle (red rectangles), holly leaf (green rectangles), granular (yellow rectangles), unsickled (blue rectangles) and uncategorized (white rectangles). (b) Snippets of representative images of healthy, SCD and SCT samples treated with $C_{\mathrm{low}}$ and $C_{\text{high}}$, respectively. The scale bar equals 10 μm in all images. A few crenated RBCs in healthy and SCT blood samples are shown in black circles. The original images are shown in Figure S3. (c) An image of a SCD sample (sample ID: D126) with manual annotation of different RBC shapes. RBC shapes shown in panel (a) are taken from this image. (d, e) Relative distribution of different RBC shapes in three SCD and three SCT blood samples treated with $C_{\mathrm{low}}$ and $C_{\text{high}}$, respectively. The horizontal lines indicate the mean values. (f) A schematic diagram summarizing the specific shapes taken by RBCs in disease and trait samples under oxygen quenching and annealing.

We treat three blood samples each of healthy, SCD and SCT blood with both $C_{\mathrm{low}}$ (0.1% SMBS) and $C_{\text{high}}$ (0.3% SMBS) for 30 min. Our aim is to visualize the shapes into which RBCs deform when the same blood sample is subjected to slow and fast Hb polymerization. Figure 3a shows some typical shapes of RBCs which include the classically sickle shape, holly leaf, granular, unsickled, and other shapes that cannot be categorized. These images of individual RBCs in Figure 3a are taken from a SCD blood sample (sample ID D126) after treating it with 0.1% SMBS for 30 min. Figure 3b shows snippets of representative images showing the resulting RBC shapes from healthy (sample ID N07), SCD (sample ID D126), and SCT (sample ID T163) blood treated with $C_{\mathrm{low}}$ and $C_{\text{high}}$. The six raw (uncropped) images from which the snippets are taken are shown along with sample IDs in Figure S3. We then ask an expert to identify and manually annotate the classically sickle‐shaped, holly leaf, granular, and unsickled RBCs in each of these six raw images. RBCs that cannot be classified into any of these four categories are marked as “uncategorized.” Figure 3c shows a typical annotated image of an SCD sample (sample ID D126) treated with $C_{\mathrm{low}}$, where RBCs of a specific shape are enclosed by rectangles of a specific color. Red rectangles enclose classically sickle‐shaped RBCs, green rectangles enclose holly leaf shapes, yellow rectangles enclose granular ones, while blue and white rectangles indicate unsickled and uncategorized RBCs, respectively.

We then count the numbers of each type of RBC in the annotated images. As shown in Figure 3d, SCD samples treated with $C_{\mathrm{low}}$, primarily exhibit a mix of classically sickle (34 ± 6%; mean ± SEM), holly leaf (37 ± 9%), and granular (18 ± 3%) RBCs. In contrast, when these samples are treated with $C_{\text{high}}$, there is a higher proportion of granular (80 ± 11%) RBCs compared to holly leaf (14 ± 7%) RBCs. Furthermore, Figure 3e demonstrates that SCT samples, when treated with $C_{\mathrm{low}}$, overwhelmingly consist of unsickled RBCs (76 ± 4%). However, when SCT samples are treated with $C_{\text{high}}$, the RBCs are primarily holly leaf‐shaped (60 ± 3%), with a smaller percentage of sickle (19 ± 2%) and granular (15 ± 3%) RBCs. Figure 3f schematically summarizes the different RBC shapes seen in SCD and SCT blood under fast and slow deoxygenation. We utilize this relative distribution of different RBC shapes in SCD and SCT samples as the basis to distinguish between SCD and SCT samples.

### 2.3 Using roundness to quantify sickle RBC shapes

“Form factor” (FF), given by Equation (1), and “roundness” (R), given by equation (2), are the most commonly used parameters for quantifying cell shapes in microscope images.^39^ Wheeless et al. explored 42 different shape descriptors, including roundness and form factor, to describe the shapes of sickled RBCs and concluded that form factor is the most suitable image analysis feature.^39^ We use ImageJ (Figure S4) to analyze images and calculate both form factor and roundness while analyzing images acquired by our smartphone microscope. It is important to note that the parameter “circularity” (C) measured by ImageJ has the same formula as the parameter form factor reported by Wheeless et al. For consistency, we use the term circularity throughout our manuscript.

(1) $$\mathrm{FF}=\frac{4\times \pi \times \text{Area}}{{\text{Perimeter}}^{2}}$$

(2) $$R=\frac{4\times \text{Area}}{\pi \times {\text{Major axix}}^{2}}$$

**FIGURE 4:**
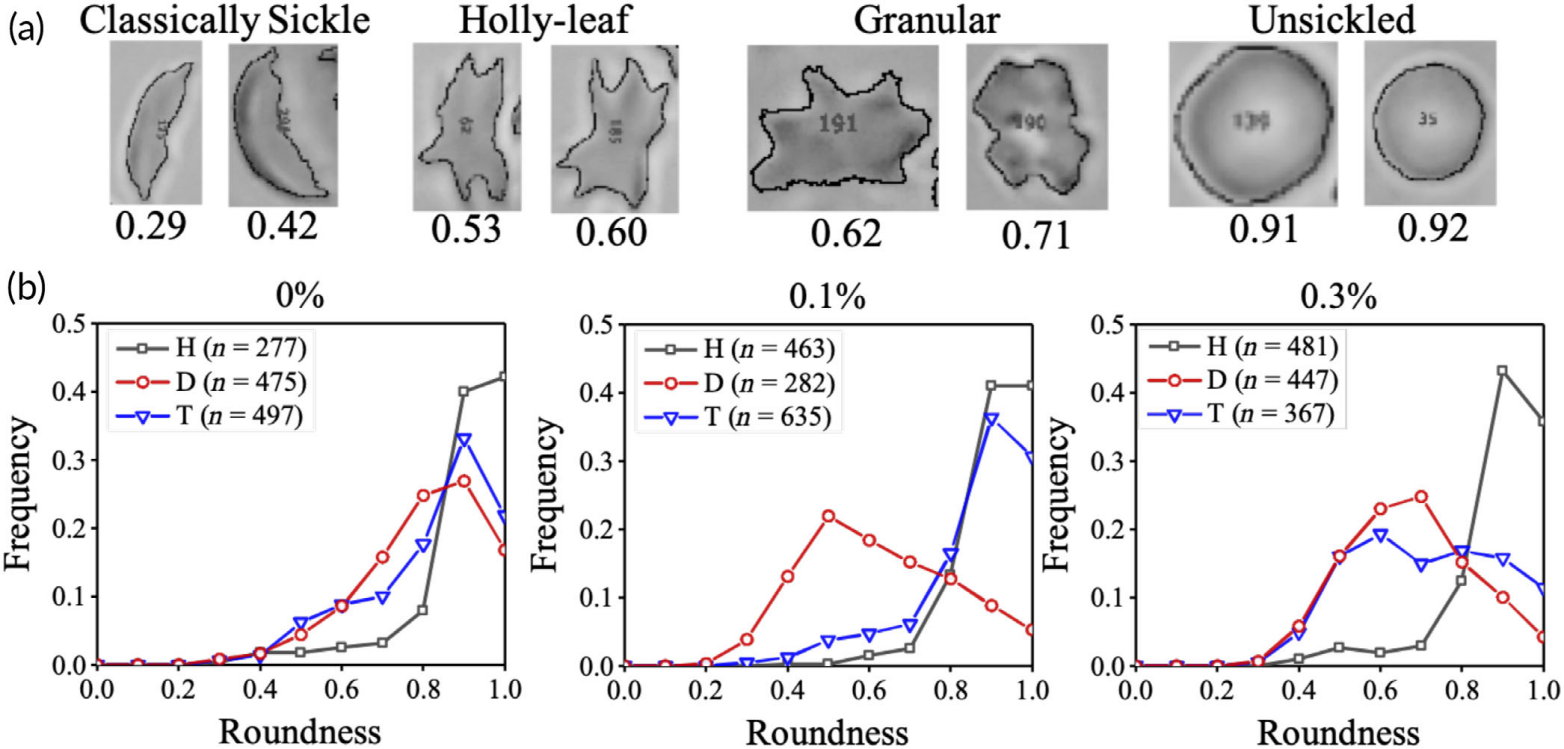
Roundness as a shape descriptor for quantifying RBC shapes in healthy and sickle blood (SCD and SCT). (a) Typical roundness values associated with different RBC shapes seen in sickle blood. (b) Roundness distributions of RBCs in healthy (H; black square), SCD (D; red circle) and SCT (T; blue triangle) samples, where the blood samples are untreated (left panel), treated with $C_{\mathrm{low}}$ (middle panel) and treated with $C_{\text{high}}$ (right panel). Each distribution is generated by pooling the data from three different blood samples (N = 3). Here, n indicates the total number of RBCs used to generate each curve.

As discussed in Figure S5, the use of circularity with our images introduces artifacts while detecting the outlines of sickle cells and wrongly classifies their shapes. Hence, we work with roundness instead. Figure 4a shows the roundness values associated with some typical sickle cell shapes.

Figure 4b displays the roundness distribution plots of RBCs in different blood samples: healthy (black square), SCD (D, red circle), and SCT (T, blue triangle). Each plot is generated by pooling data from three blood samples. All roundness plots are normalized such that the area under each curve is unity. In the left panel, where there is no presence of SMBS, the distribution of healthy blood peaks at 1.0. This indicates that biconcave RBCs appear circular in shape. Similarly, the distributions for SCD and SCT samples also peak at 0.9, as most RBCs retain their biconcave shape without the influence of SMBS. The middle panel shows the roundness distributions of blood treated with $C_{\mathrm{low}}$ (0.1% SMBS). The roundness distribution of healthy RBCs remains unchanged since they are not affected by SMBS. However, in SCD blood, the roundness distribution peaks at 0.5. In SCT blood treated with the same low concentration of SMBS, most RBCs do not sickle, resulting in a roundness distribution peak similar to that of healthy blood (0.9). The right panel illustrates the roundness distributions of RBCs treated with $C_{\text{high}}$ (0.3% SMBS). In SCD blood, the roundness distribution peaks at 0.7, while in SCT blood, it peaks at 0.6. As anticipated, the distribution for healthy RBCs treated with the high concentration of SMBS has a peak at 0.9. Due to similar roundness values between RBCs with granular and holly leaf shapes, the roundness distributions of SCD and SCT samples treated with the high concentration of SMBS overlap. Similarly, the peaks of the distributions for healthy and SCT samples treated with the low concentration of SMBS also overlap. These results show that the comparison of two roundness distributions at 0.1% and 0.3% SMBS is necessary for distinguishing between SCD, SCT, and healthy blood samples.

### 2.4 A shape‐based classifier is developed to distinguish between healthy, SCD and SCT blood samples

Since all roundness plots in Figure 4b are normalized such that the total area under each curve equals unity, the only way to distinguish them is by measuring the area between *two specific roundness values*. Since the roundness distributions for healthy and SCT overlap for slow deoxygenation and the roundness distributions for SCD and SCT overlap for fast deoxygenation, it is not possible to distinguish all three samples by considering the area under the curves in just one region. Therefore, we consider the areas for two separate ranges of roundness values and call these two areas $P_{1}$ and $P_{2}$ (violet and green rectangles shown in Figure S6A), respectively. It allows the shape information in an image to be denoted by two coordinates $P_{1}$ and $P_{2}$ in the $\left(P_{1},P_{2}\right)$ parameter space. The problem of separating sickled and unsickled blood under a specific SMBS concentration is now simplified to plotting the coordinates of each sample in the (*P*_1_, *P*_2_) plane, clustering them, and finding a linear classifier that separates the sickled and unsickled regions.

Next, we need to identify two roundness ranges corresponding to *P*_1_ and *P*_2_ such that the sickled and unsickled clusters are optimally separated. We vary both the width of the roundness range (from 0.1 to 0.5) and the starting position of the adjacent roundness ranges. It results in 16 possible combinations given by Table S3. Applying *k*‐means (*k* = 2) clustering algorithm, and analyzing the connectivity (with 10 nearest neighbors) and Dunn index of the resulting clusters for each of the 16 combinations, we identify the two most optimal combinations of roundness ranges corresponding to the most optimum $P_{1}$ and $P_{2}$ combinations. Combination #10 spans roundness values from 0.2 to 0.8, with $P_{1}$ indicating the area between roundness values of 0.2 and 0.5, while $P_{2}$ indicating the area between roundness values of 0.5 and 0.8. On the other hand, combination #12 spans roundness values from 0.4 to 1.0 with $P_{1}$ ranging from 0.4 to 0.7 and $P_{2}$ ranging from 0.7 to 1.0. Combination #10 includes information about sickle RBCs (0.2 < *R* < 0.4) and excludes unsickled RBCs (*R* >0.8). Combination #12 lacks information about sickle RBCs (*R* < 0.4) but includes unsickled RBCs (*R* >0.8). Consequently, we proceed with combination #10 for further analysis. See Figures S6–S8 and Table S3 for details.

**FIGURE 5:**
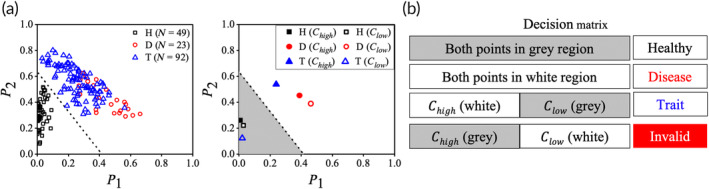
Development of a classifier from our training dataset. (a) We develop a classifier (dotted line) from a training dataset consisting of 164 blood samples. It separates the parameter space into two regions. (b) The decision matrix illustrates how the position of the two points on the classifier, representing $C_{\text{high}}$ and $C_{\mathrm{low}}$ images, determines whether an unknown blood sample is classified as healthy, SCD, or SCT.

Figure 5a displays our training dataset, consisting of 164 samples with known hemoglobin profiles, plotted in the $P_{1}–P_{2}$ parameter space using combination #10. We utilize a support vector machine (SVM) model with a linear kernel and a cost of 10 on the raw data on this dataset to create a classifier given by the equation: *y* = −1.527*x* + 0.633 (dotted line). The classifier differentiates between healthy and sickle clusters (including both SCD and SCT). To generate the training dataset, we treat SCD samples with only 0.1% SMBS, while both SCT and healthy samples are treated with only 0.3% SMBS. In Figure 5b, our decision matrix based on the classifier is presented. The points corresponding to both $C_{\mathrm{low}}$ and $C_{\text{high}}$ for healthy samples (hollow and solid black squares, respectively) are located in the gray region. The points representing SCD samples (hollow and solid red circles, respectively) are situated in the white region. For a SCT sample, the point representing $C_{\mathrm{low}}$ (hollow blue triangle) is in the gray region, while the point representing $C_{\text{high}}$ (solid blue triangle) is in the white region. However, if the point representing $C_{\mathrm{low}}$ falls in the white region and the point representing $C_{\text{high}}$ falls in the gray region, the analysis is deemed invalid, and the experiment needs to be repeated.

### 2.5 Validation of the classifier in a pilot study with 35 unknown blood samples

**FIGURE 6:**
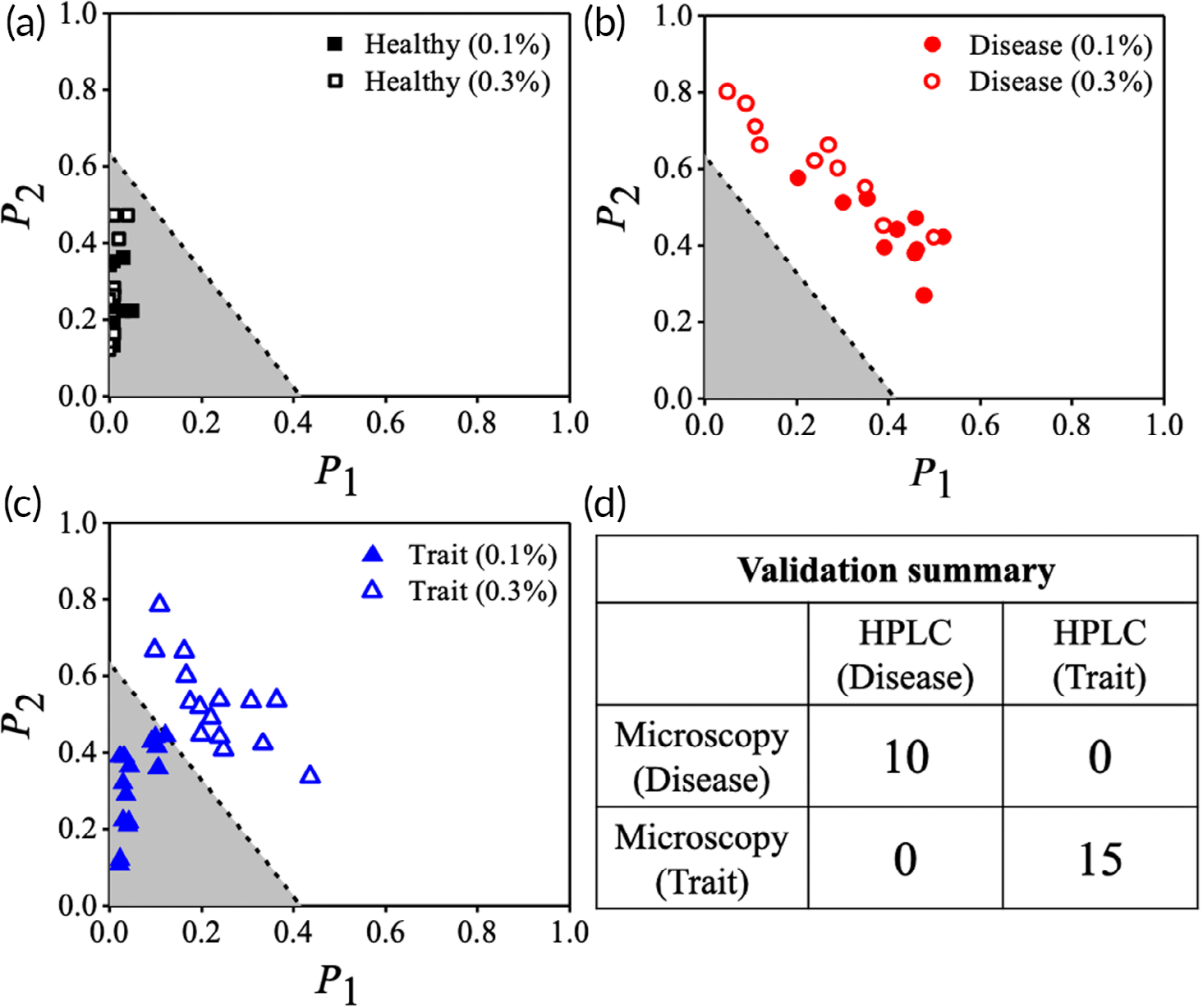
Pilot validation of our classifier with 35 unknown blood samples. We accurately classified 10 healthy (a), 10 SCD (b), and 15 SCT (c) samples. (d) The diagnosis from microscopy shows 100% concordance with HPLC data for the classifier, with no false positive or false negative diagnosis.

In a proof‐of‐concept study, we successfully validate the ShapeDx™ protocol using a small group of 35 unidentified blood samples. Out of these samples, 10 are identified as healthy, 10 as SCD, and 15 as sickle cell trait (SCT) (Figure 6a–c). Additionally, the same blood samples are analyzed using HPLC in a blinded manner. Our results match the HPLC results perfectly, with 100% concordance. The table in Figure 6d demonstrates that there are no instances of false positives (identifying SCT samples as SCD) or false negatives (identifying SCD samples as SCT) among the sickle blood samples we test in this pilot study. This proof‐of‐concept study represents a significant milestone as it establishes that the shape of RBCs can accurately differentiate between sickle cell homozygous (SCD) and heterozygous (SCT) blood samples. However, it is important to perform the test in presence of other hemoglobin variants as confounding factors and with blood samples obtained from newborns with high levels of fetal hemoglobin.

## 3 DISCUSSION

ShapeDx™ (Figure 1) exploits the differential hypoxia generated using *two* concentrations of an oxygen scavenger on the same blood sample to distinguish between SCD and SCT blood by microscopy. We can create a controlled hypoxic environment in a 10 μL volume of blood by simply controlling the SMBS concentration within an imaging microchamber. It reduces the sickling time of both SCD and SCT blood to just 30 min compared to bulk deoxygenation assays that take several hours (Figure 2). The optimized dimensions of the imaging microchamber facilitate correct orientation of RBCs during imaging, while ensuring 150–200 RBCs are seen in the field of view. Using these controlled sickling assays, we demonstrate the relationship between different hemoglobin polymerization kinetics and resulting RBC shapes in the same blood sample (Figure 3). Subjecting SCD blood to slow polymerization leads to a mix of classically sickle, holly leaf and granular RBCs,^27^, ^29^, ^32^ whereas fast polymerization leads to largely granular RBCs.^32^, ^39^ There is almost no change in RBC shape when SCT blood is subjected to slow polymerization owing to the combined effect of fewer nucleation sites, HbAS heterodimers, and slow deoxygenation.^35^, ^36^ In contrast, subjecting SCT blood to fast polymerization results in primarily holly leaf‐shaped RBCs. The varying shapes of RBCs are reflected in the roundness distribution profiles of different blood types under different rates of deoxygenation. Disease, trait, and healthy blood samples show distinct roundness profiles under different concentrations of SMBS. Figure 4 shows that the roundness profiles of disease samples are very different from those of healthy or trait samples under slow deoxygenation. Similarly, the roundness profiles of healthy samples are very different from those of disease or trait samples under fast deoxygenation. Hence, we use the roundness distributions of RBCs under slow and fast hypoxia to develop a classifier (Figure 5) capable of separating healthy, disease, and trait samples, and validate the classifier using 35 blood samples (Figure 6).

ShapeDx™ requires only a drop of blood instead of the typical 2–4 mL of venous blood needed for HPLC, thereby reducing the blood loss experienced by anemic patients. We choose a simple design for the imaging microchamber and fabricate it using inexpensive components outside a cleanroom. To conduct the test in remote field locations, we develop a robust and portable smartphone microscope capable of capturing high‐quality brightfield images of unstained RBCs. The smartphone's GPS feature can record the location of new patients. For image analysis, we employ open‐source software ImageJ and R, facilitating open research. Unlike existing reports, the images captured using our microscope can directly be processed without any image transformation or pre‐processing. In spite of a few image artifacts (e.g., biconcave RBCs lying sideways, crenated RBCs, two or more overlapping RBCs, etc.), our workflow is very robust. Due to fast (<60 min) turnaround time, our test can enhance patient compliance with screening efforts.^41^ We tested ShapeDx™ in two field locations to demonstrate its robustness. We also successfully trained a layperson to visually compare the two images and identify an unknown blood sample as healthy or sickle, demonstrating the potential of ShapeDx™ as a screening test. A shortcoming of ShapeDx™ is the short shelf‐life of the SMBS and RPMI solutions.

The key strength of ShapeDx™ lies in its robustness and simplicity, making it a potential gamechanger to diagnose sickle cell anemia in resource‐limited settings. Our future work involves testing ShapeDx™ with newborn samples and on individuals undergoing blood transfusion or hydroxyurea treatment. Incorporation of crossed thin‐film polarizers into our imaging setup can lead to better visualization of Hb polymerization. We also plan to investigate how RBC shape correlates with disease severity in SCD and SCT.

## 4 METHODS

### 4.1 Blood collection

The study is approved by the Institute Ethics Committee (IEC) of IIT Bombay with approval numbers IITB‐IEC/2016/016, IITB‐IEC/2017/020, and IITB‐IEC/2018/042. We use leftover blood samples collected during sickle cell screening camps organized by Shirin and Jamshed Guzder Regional Blood Centre (Valsad, Gujarat) and Dayanand Hospital (Talasari, Maharashtra) after obtaining written informed consent from participants. The participants received medical assistance following the existing standard of care. Results of this study were neither made available to the participants, nor included in clinical decision making. Samples are provided in a gender‐blind manner as sickle cell anemia is an autosomal recessive disease with an equal chance of inheritance in both males and females. We exclude blood samples of those individuals who (i) are under treatment with folic acid or hydroxyurea, or (ii) received blood transfusion within 3 months prior to blood collection. HPLC performed during regular screening camps is used as the gold standard test for comparison. We store all blood samples at 4°C in K3/EDTA vacutainer tubes prior to use and perform all microscopy tests within 48 h of blood collection.

### 4.2 Choice of diluent and dilution factor

**FIGURE 7:**
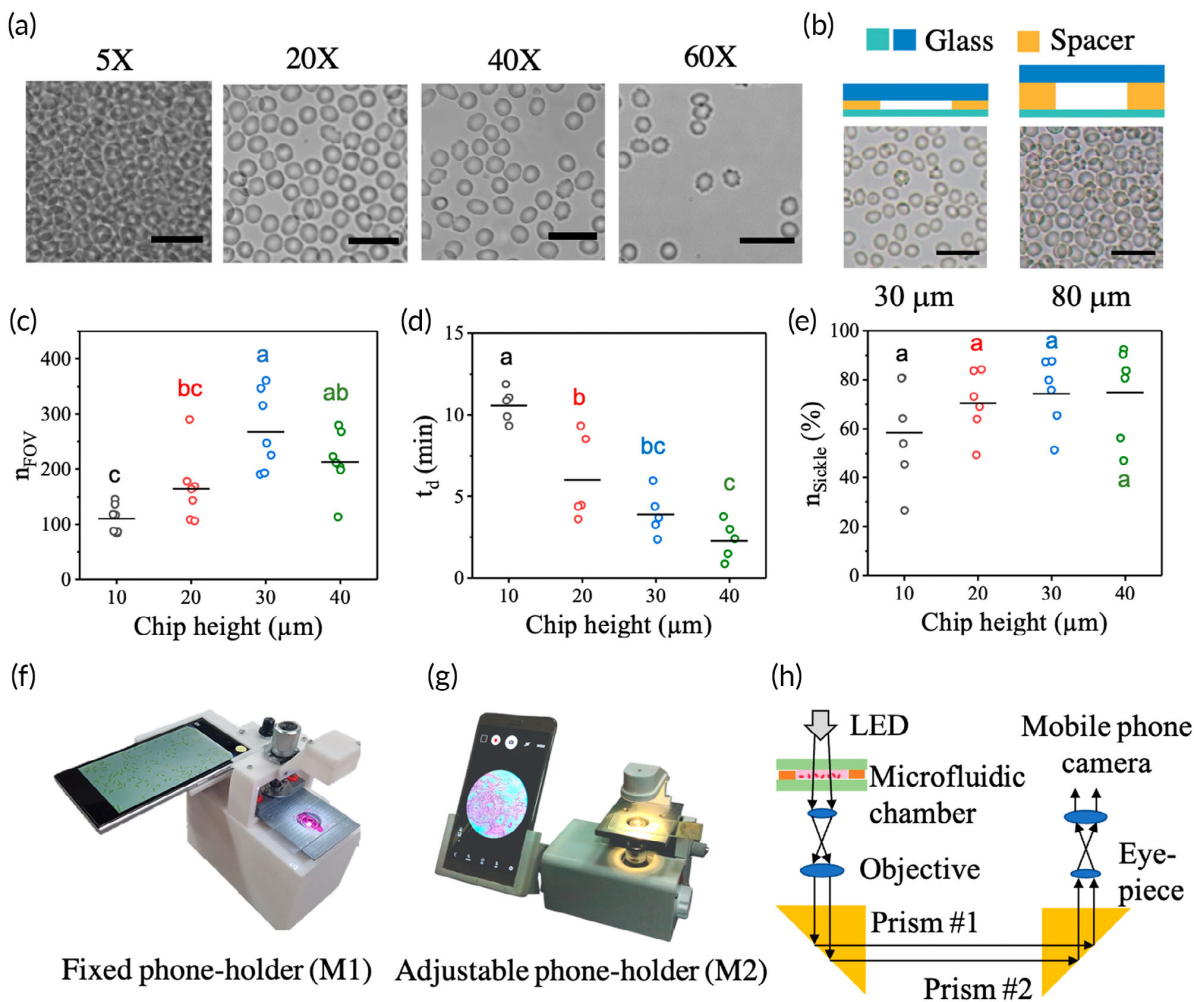
Our experimental set‐up. (a) Optimization of blood dilution. (b) RBCs inside 30 μm chamber lie flat, while RBCs are stacked in random orientations in the 80 μm chamber. The scale bar equals 20 μm in all images. Optimization of the chamber height in terms of (c) the number of healthy RBCs in the field of view ($n_{\mathrm{FOV}}$), (d) the time taken ($t_{d}$) for the first RBC in the field of view to start sickling, and (e) the percentage of sickle cells ($n_{\text{sickle}}$) at *t* = 30 min. The horizontal lines indicate the mean values in panels (c–e). (f, g) Photos of the smartphone microscope models with fixed phone holder (M1) and adjustable phone holder (M2), respectively. (h) Light path inside our smartphone microscope.

We test 0.9% normal saline (NS), 5% dextrose, 1X phosphate buffered saline (PBS) and cell culture media (RPMI‐1640) as diluents to identify the one which imparts minimal stress to RBCs and avoids crenation. Dextrose leads to clumping of RBCs even in healthy blood samples, while sickling in PBS and NS takes 2–3 times longer compared to RPMI‐1640. Hence we dilute blood in RPMI‐1640. After testing 5X, 20X, 40X, and 60X dilutions, we choose 20X as the dilution factor as there were >200 RBCs in the field of view without stacking (Figure 7a). RPMI‐1640 should be refrigerated at 4°C.

### 4.3 Preparation of SMBS

We use SMBS, ranging in concentration from 0.1% to 0.5%, as the chemical sickling agent in this study. SMBS solutions are freshly prepared in RPMI‐1640 before each experiment and discarded after 6 h. Prior to use, it is stored in an inert environment to preserve its reducing activity and avoid confounding results.

### 4.4 Preparation of imaging micro‐chamber

We fabricate a simple 10 mm × 22 mm imaging chamber using a glass plate and a cover slip separated by a spacer. Figure S9 has details of fabrication of the imaging microchamber. Glass is chosen due to its good optical properties and non‐permeability to oxygen, which promotes fast sickling. We obtain different chamber heights (e.g., 10, 20, 30, 40, 80, and 100 μm) using double‐sided adhesive films of specific thickness as the spacer. While increasing the chamber height (≥80 μm) leads to stacking of RBCs (Figure 7b), an increase in confinement leads to crenation. Therefore, we focus on chips of intermediate heights, for example, 10, 20, 30, and 40 μm. Figure 7c shows the number of RBCs ($n_{\mathrm{FOV}}$) in the field of view inside chambers of different heights. While fewer RBCs are present inside 10 μm (110 ± 10; mean ± SEM; n = 7) and 20 μm (165 ± 23) chambers, the image processing algorithm detects more than 200 RBCs inside 30 μm (268 ± 27) and 40 μm (213 ± 21) chambers. As there are more densely packed RBCs inside the 40 μm high chamber, these are eliminated as clusters by the image processing algorithm. This results in a decrease in the number of RBCs counted by the algorithm inside the 40 μm high chamber. As shown in Figure 7d, we treat five SCD samples with 0.1% SMBS and measure the delay time ($t_{d}$). The values of $t_{d}$ are 10.6 ± 0.5 min (mean ± SEM, n = 5), 6.0 ± 1.2 min, 3.9 ± 0.6 min and 2.3 ± 0.5 min for chip heights of 10, 20, 30 and 40 μm, respectively. We also measure the percentage of sickled RBCs $\left(n_{\text{sickle}}\right)$ at t = 30 min in these samples. As shown in Figure 7e, the mean value of $n_{\text{sickle}}$ ranges from 58% to 75% with a large variability in the data. We find that there are >250 RBCs inside a 30 μm chamber with minimal crenation and fast sickling ($t_{d}$ = 3.9 ± 0.6 min). Therefore, we optimize the height of the imaging microchamber to be 30 μm.

### 4.5 Sickling experiment

Two separate drops (~5 μL) from each blood sample are taken for the sickling experiment. One drop is mixed with 95 μL of 0.1% SMBS and another drop is mixed with 95 μL of 0.3% SMBS to obtain 20X diluted blood. The pH of blood mixed with 0.1% and 0.3% SMBS are 6.05 ± 0.2 and 5.83 ± 0.1 (mean ± SEM), respectively. No hematocrit adjustment of blood is performed so as to mimic the typical conditions of a screening camp. Ten microliters of each blood and SMBS mix is loaded into an imaging microchamber. Two open sides of the chip are sealed with fast‐drying clear nail lacquer and the two chips are set aside for 30 min at room temperature to allow differential hypoxia to take over. A chip is discarded if air bubbles are trapped during loading of blood or if the RBCs are crenated immediately after adding blood. In that case the experiment is repeated with a new chip.

### 4.6 Development of a portable digital microscope

We developed a portable digital phone microscope in‐house, with two versions M1 and M2, to be used at the field (Figure 7f,g). It is a battery‐operated inverted transmission microscope with 40X (0.65 NA) air objective and a total optical magnification of 600X. Figure 7h shows the light path inside the microscope. The microscope has a 1 W white LED, fitted with a collimating lens that acts as the illumination source. The light transmitted through the sample passes through a 40X (0.65 NA) air objective and two right‐angled prisms to bend the light path by 180°. The beam then passes through a 15X eye‐piece lens and is focused to form an image on the smartphone camera. A total optical magnification of 600X is achieved over a tube length of 160 mm. We develop two microscope models, one with a fixed phone holder (M1; Figure 7f) and another where the phone holder can be tilted by any angle for viewing comfort (M2; Figure 7g). Figures S10 has detailed diagrams showing the optical and mechanical parts of the portable digital microscope.

### 4.7 Imaging and image classification

We capture brightfield images of unstained RBCs for diagnosis. While we also capture real‐time videos (Movies [Link], [Link]) of the sickling process at 30 fps to understand the sickling kinetics, these videos do not play any role in our diagnostic workflow. The images are analyzed by ImageJ.^42^ For the microscope and the mobile phone combination, 6 pixels equals 1 μm. After filtering and segmenting the image, we measure roundness and solidity of each RBC in the field of view. RBCs with solidity <0.8 are excluded from analysis as these correspond to partially focused RBCs or RBCs lying sideways. Roundness values of remaining ~150–200 RBCs in the field of view are binned into 10 groups with 0.1 width to obtain the roundness distribution. Each distribution is normalized to the total number of RBCs included in the analysis.

### 4.8 Statistical methods

Data acquired in this study are reported as mean of three or more samples or experiments. All statistical analyses are carried out using R software and RStudio. Data are analyzed by parametric one‐way ANOVA followed by Tukey's post hoc test for multiple comparisons. Statistical significance is set at 95% confidence level for all tests (*p* < 0.05).

## AUTHOR CONTRIBUTIONS

**Claudy D'Costa:** Conceptualization (equal); data curation (equal); formal analysis (equal); investigation (equal); methodology (equal); software (equal); validation (equal); visualization (equal); writing – original draft (equal); writing – review and editing (equal). **Oshin Sharma:** Conceptualization (equal); data curation (equal); formal analysis (equal); investigation (equal); methodology (equal); validation (equal); visualization (equal); writing – original draft (equal); writing – review and editing (equal). **Riddha Manna:** Conceptualization (equal); formal analysis (equal); methodology (equal); software (equal); visualization (equal); writing – original draft (equal); writing – review and editing (equal). **Minakshi Singh:** Data curation (equal); investigation (equal); methodology (equal); visualization (equal); writing – review and editing (equal). **Samrat Singh:** Data curation (equal); methodology (equal). **Srushti Singh:** Data curation (equal); formal analysis (equal); investigation (equal); methodology (equal). **Anish Mahto:** Data curation (equal); investigation (equal). **Pratiksha Govil:** Data curation (equal); formal analysis (equal); investigation (equal); methodology (equal); writing – original draft (equal); writing – review and editing (equal). **Sampath Satti:** Data curation (equal); formal analysis (equal); methodology (equal); visualization (equal); writing – review and editing (equal). **Ninad Mehendale:** Investigation (supporting); methodology (supporting). **Yazdi Italia:** Investigation (equal); resources (equal). **Debjani Paul:** Conceptualization (equal); formal analysis (equal); funding acquisition (equal); methodology (equal); project administration (equal); resources (equal); supervision (equal); writing – original draft (equal); writing – review and editing (equal).

## CONFLICT OF INTEREST STATEMENT

Two Indian patent applications have been filed for the portable microscopes, which are licensed to MedPrime Technologies. Samrat, one of the authors of this manuscript, is the CEO and co‐founder of MedPrime Technologies. An Indian patent and an Indian trademark application have been filed for the diagnostic test referred to as ShapeDx™ in this manuscript. Both the patent and the trademark are licensed to Lords Mark Technologies Pvt. Ltd. Neither MedPrime Technologies nor Lords Mark Technologies Pvt. Ltd have contributed financially to the project or have had any influence over the research direction. The remaining authors declare that the research was conducted in the absence of any commercial or financial relationships that could be construed as a potential conflict of interest.

### PEER REVIEW

The peer review history for this article is available at https://www.webofscience.com/api/gateway/wos/peer-review/10.1002/btm2.10643.

## Supporting information


**Data S1.** Supporting information.

**Movie S1.** Sickling video of a disease sample treated with 0.1% sodium metabisulphite. The 30 min video has been sped up 29.5 times. The sample ID is D188.

**Movie S2.** Sickling video of a disease sample treated with 0.3% sodium metabisulphite. The 30 min video has been sped up 29.8 times. The sample ID is D188.

**Movie S3.** Sickling video of a trait sample treated with 0.1% sodium metabisulphite. The 30 min video has been sped up 29.5 times. The sample ID is T187.

**Movie S4.** Sickling video of a trait sample treated with 0.3% sodium metabisulphite. The 30 min video has been sped up 29.5 times. The sample ID is T187.

## Data Availability

The main data from which conclusions are drawn are included in the manuscript and the Supporting Information. Suitably de‐identified images of blood samples are available for research and teaching purposes from the corresponding author on request. All codes used for analyzing images and processing the data can be found at the following link. https://github.com/ridz46/SickleCellDataAnalysis.
