# Supplementary material for: Differential sensitivity to hypoxia enables shape‐based classification of sickle cell disease and trait blood samples at point of care
Source: Bioeng Transl Med. 2023 Dec 27;9(4):e10643. doi: 10.1002/btm2.10643 (PMC11256192; doi:10.1002/btm2.10643)
Supplement: Supplementary file 1 — Data S1. Supporting information. [file BTM2-9-e10643-s001.docx]

**Supporting information**

**Differential sensitivity to hypoxia enables shape-based classification of sickle cell disease and trait blood samples at point of care**

Claudy D’Costa, Oshin Sharma, Riddha Manna, Minakshi Singh, Samrat, Srushti Singh, Anish Mahto, Pratiksha Govil, Sampath Satti, Ninad Mehendale, Yazdi Italia and Debjani Paul

1. **Comparison of ShapeDx with existing POC tests**

***Table T1****: Features of various upcoming POC tests*

The table T1 compares the existing point-of-care (POC) tests with ShapeDx. The reference numbers refer to the reference list in the main manuscript.

**2. Hemoglobin polymerization and resulting RBC shapes**

**
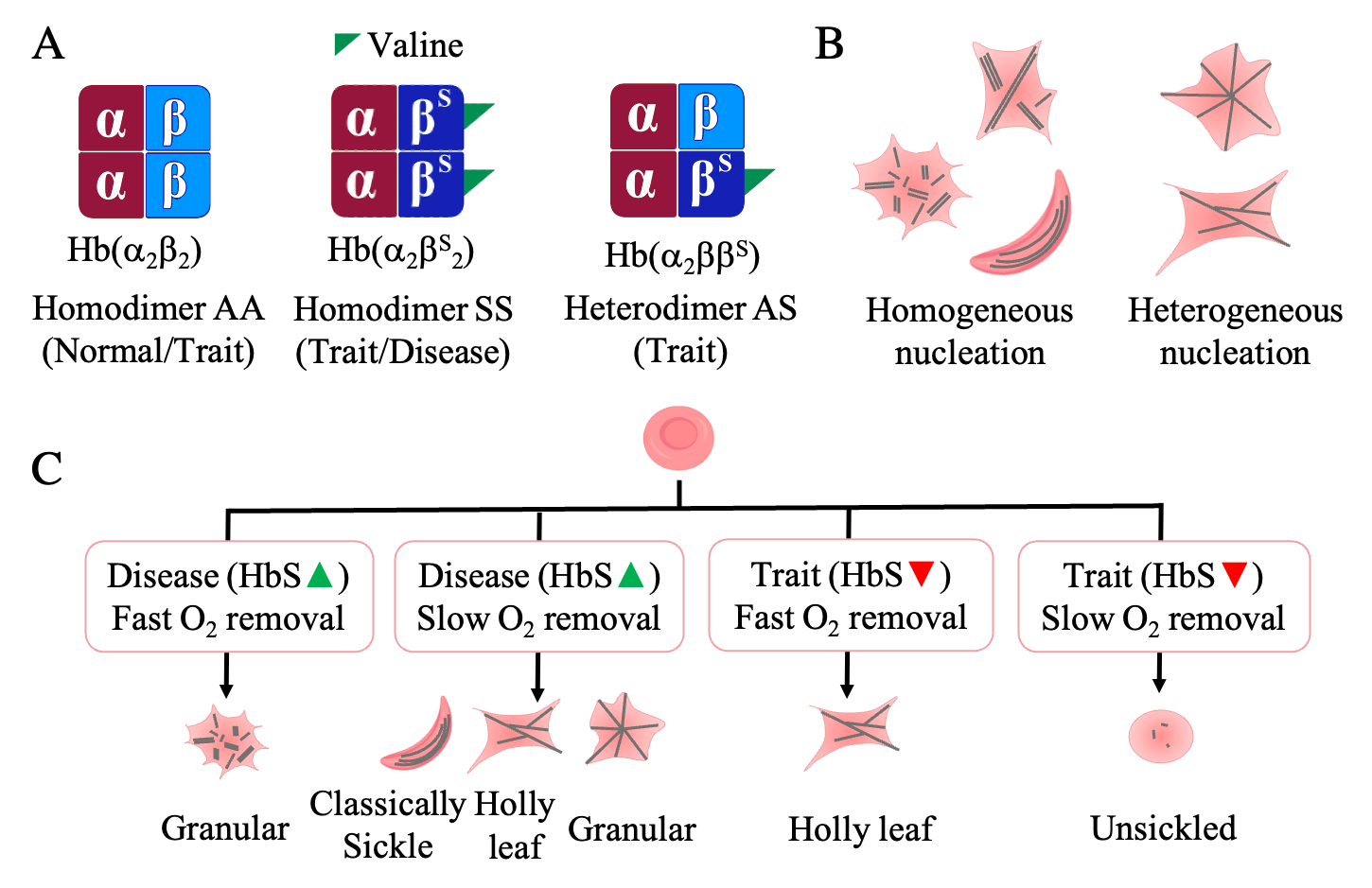
**

***Figure S1.*** *Schematic diagram relating hemoglobin polymerization to shapes of sickled RBCs. (A) Normal hemoglobin exists as homodimer AA (*α_2_β_2_*). This homodimer is also partially found in sickle trait blood. Sickle hemoglobin can form either a homodimer SS* (α_2_β*^S^*_2_*;*  *found in both trait and disease blood) or a heterodimer AS (*α_2_ ββ*^S^) with normal hemoglobin (found only in trait blood). (B) During hemoglobin polymerization, homogeneous nucleation leads to one or more non-branched polymer chains, while heterogeneous nucleation leads to branched polymers. The kind of hemoglobin polymerization that takes place inside a RBC affects its shape. (C) A pictorial summary of how shapes of sickled RBCs depend on HbS concentration and deoxygenation rate. Green ‘up triangle’ symbol indicates a high HbS concentration or fast deoxygenation. Red ‘down triangle’ symbol indicates low HbS concentration or slow deoxygenation.*

The total hemoglobin in homozygous sickle blood (SCD) consists of 95% - 98% HbS, 2% - 3% HbA2 (another variant of normal hemoglobin) and 2% HbF (fetal hemoglobin). Heterozygous sickle cell blood (SCT) contains 30% - 40% HbS, 55% - 65% HbA, 2% - 3% HbA2 and ~ 2% HbF (6). Normal hemoglobin (HbA) consists of two alpha-globin (α2) and two beta-globin (β2) subunits. As shown in **figure S1A**, blood of healthy individuals (i.e. those without the sickle gene) has HbAA homodimers (α2β2). The sickle mutation replaces one of the hydrophilic glutamic acid residues in the beta subunit by a hydrophobic valine residue, which acts as a polymerization site. Under low oxygen concentrations, the valine residues can form hydrophobic bonds with each other, leading to the polymerization of the HbS molecules and forming rigid polymer rods. Replacement of a single normal beta subunit (β) by a sickle beta subunit (β^s^) can lead to the formation of sickle heterodimers (HbAS), while the replacement of both beta subunits leads to the formation of sickle homodimers (HbSS). SCD blood has HbSS homodimers (α2βS2). SCT blood contains a mix of HbAA homodimers (α2β2), HbSS homodimers (α2βS2) and HbAS heterodimers (α2 ββS).

According to the ‘double nucleation model’ proposed by Ferrone *et al*, hemoglobin polymerization can proceed by homogenous or heterogeneous nucleation (**figure S1B**) (25). Homogeneous nucleation involves growth of independent polymer chains from HbS molecules in solution, whereas heterogeneous nucleation involves formation of branches on already existing polymers. Even though homogeneous nucleation is thermodynamically less favourable, Ferrone and others suggested that it can be sustained in samples with high initial HbS concentration for a longer duration before heterogeneous nucleation takes over. Since SCD blood has high HbS concentration, it follows that these samples are likely to sustain homogeneous nucleation for longer durations. The authors further observed that fast deoxygenation leads to more homogeneous nucleation sites and formation of randomly oriented polymer chains. Hence, we expect that faster deoxygenation in SCD samples will lead to more granular RBCs, and very few, if any, sickle-shaped RBCs. Slow deoxygenation in these samples would lead to sickle RBCs resulting from sustained homogeneous nucleation, as well as some holly leaf and granular RBCs formed due to heterogeneous nucleation.

In addition to HbS concentration and the rate of deoxygenation, HbS polymerization in SCT samples is also affected by the presence of HbAS heterodimers which can constitute as much as ~ 49% mole fraction of the total haemoglobin (23). HbS concentration in SCT samples is too low to sustain homogeneous nucleation. Moreover, HbAS heterodimers have a lower probability to take part in polymerization compared to HbSS homodimers (24). Under slow deoxygenation, due to a lack of nucleation sites, RBC morphology in trait blood appears to be unchanged. When deoxygenation is rapid, trait RBCs deform into primarily holly leaf shapes resulting from heterogeneous nucleation. **Figure S1C** summarizes the combined effect of HbS concentration and rate of deoxygenation on RBC shapes.

**3. Obtaining the deoxygenation time for different concentrations of sodium metabisulphite**

***Table T2****: Fitting parameters to extract the decay time constant (τ) describing the decrease in dissolved oxygen present in RPMI-1640 for different concentrations of the oxygen scavenger sodium metabisulphite.*

| *Sodium metabisulphite* | 0.1% | 0.2% | 0.3% | 0.4% | 0.5% |
| --- | --- | --- | --- | --- | --- |
| *A* | 112.5 | 117.1 | 106.6 | 100.7 | 99.6 |
| *B* | - 7.2 | - 4.5 | - 0.9 | 0.2 | 0.8 |
| *τ (min)* | 12.2 | 9.3 | 6.1 | 3.5 | 1.6 |

**
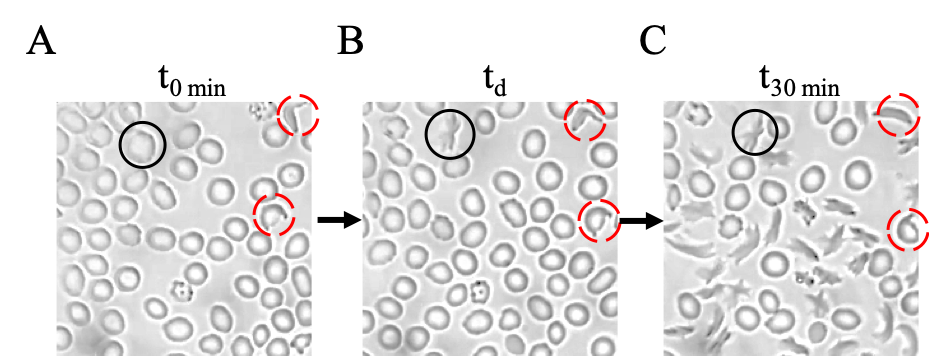
4. Delay time**

***Figure S2.*** *Measurement of delay time (*$t_{d}).$ *Delay time is the time taken for the first unsickled RBC in the entire field of view to start sickling. The image in panel A is captured at time* $t_{0 min}$*, which indicates the frame captured immediately after adding SMBS. The image in panel B shows that the RBC enclosed by the black circle has just started sickling. This time point is indicated as* $t_{d}$*. No other RBC in this frame has started sickling yet. Panel C shows a frame captured 30 min after adding SMBS. Most RBCs in the frame are sickled by this time. The two RBCs inside the dashed red circles are already sickled when we start imaging, and therefore, are not considered for determining* $t_{d}$*.*

**5. Raw images of disease, trait and healthy samples treated with sodium metabisulphite for 30 min**

**
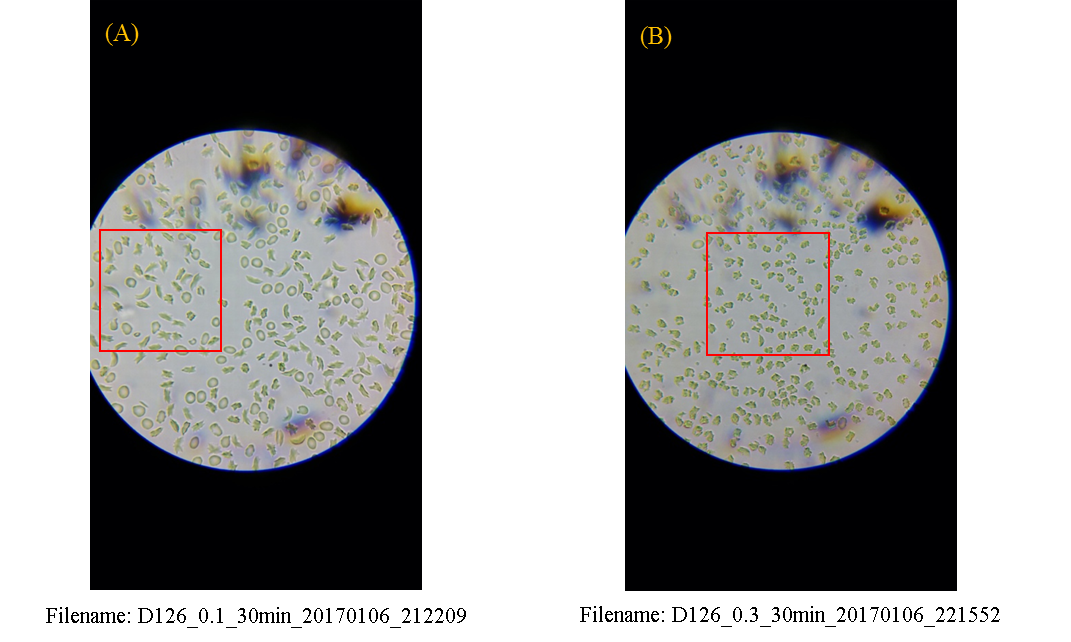
Figure S3** shows some representative raw images of disease, trait and healthy blood samples. The red rectangles show the areas that were used as snippets in figure 4 of the manuscript.

*
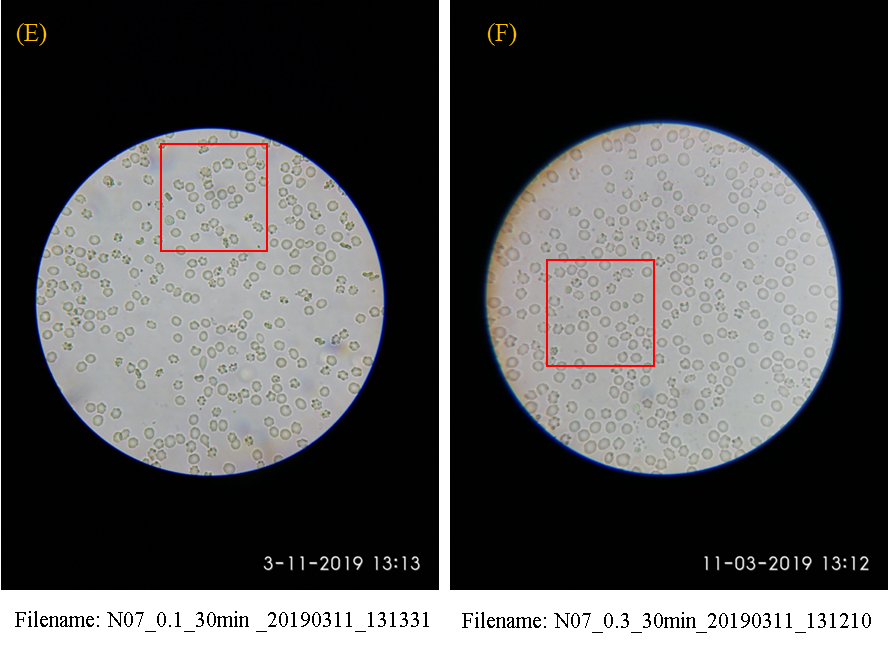
***
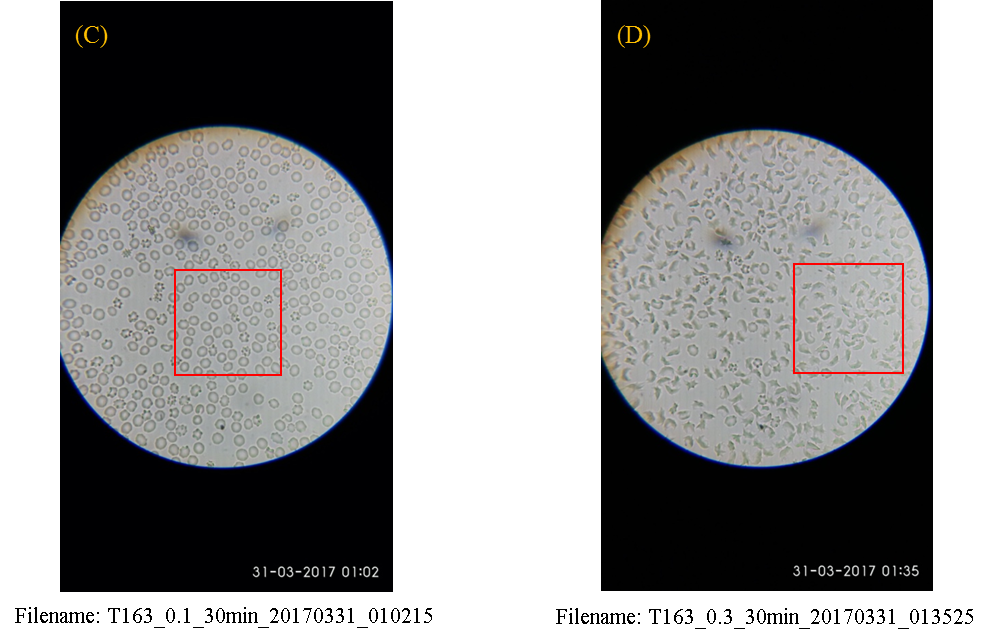
**

***Figure S3.*** *Raw images of disease (A and B), trait (C and D) and healthy (E and F) samples treated with 0.1% (left column) and 0.3% (right column) sodium metabisulphite respectively. The red rectangles show the areas from which snippets were taken for figure 3A.*

**6. Workflow for image analysis of RBCs**

RGB images captured by the smartphone are converted into 8-bit grayscale images. After background subtraction and automatic thresholding, morphological operations are performed to obtain the outlines of the RBCs. Using an area filter, we rule out RBCs with areas <500 pixels (~2.5 μm^2^) and > 7500 pixels (~9.8 μm^2^) as possible debris and clusters of RBCs respectively. The detailed steps in ImageJ are described below.

The captured image **(1)** is first cropped **(2)** to fit the field of view and converted into an 8-bit grayscale image **(3).** We then subtract the background with a rolling ball radius of 50 pixels **(4).** Next, we make the grayscale image into a binary one using the automatic thresholding algorithm of ImageJ. The RBCs appear black on a white background **(5).** This binary image is them converted into a mask, to make the RBCs appear white against a black background **(6)**.

On the masked binary image, we perform the operation ‘fill holes’ **(7)**. This is required as the central part of the biconcave RBCs remain out of focus. We then analyze the particles (RBCs) to get a list of unique identifiers for each RBC and their shape descriptors, including roundness and solidity. Next, we apply an area filter described earlier to exclude debris and connected cells (**8a**). The panel **8b** shows the same cells without the area filter. We then apply a solidity cut-off, where all RBCs with solidity <0.8 are excluded. The RBCs remaining after this step are used to plot the roundness distributions.


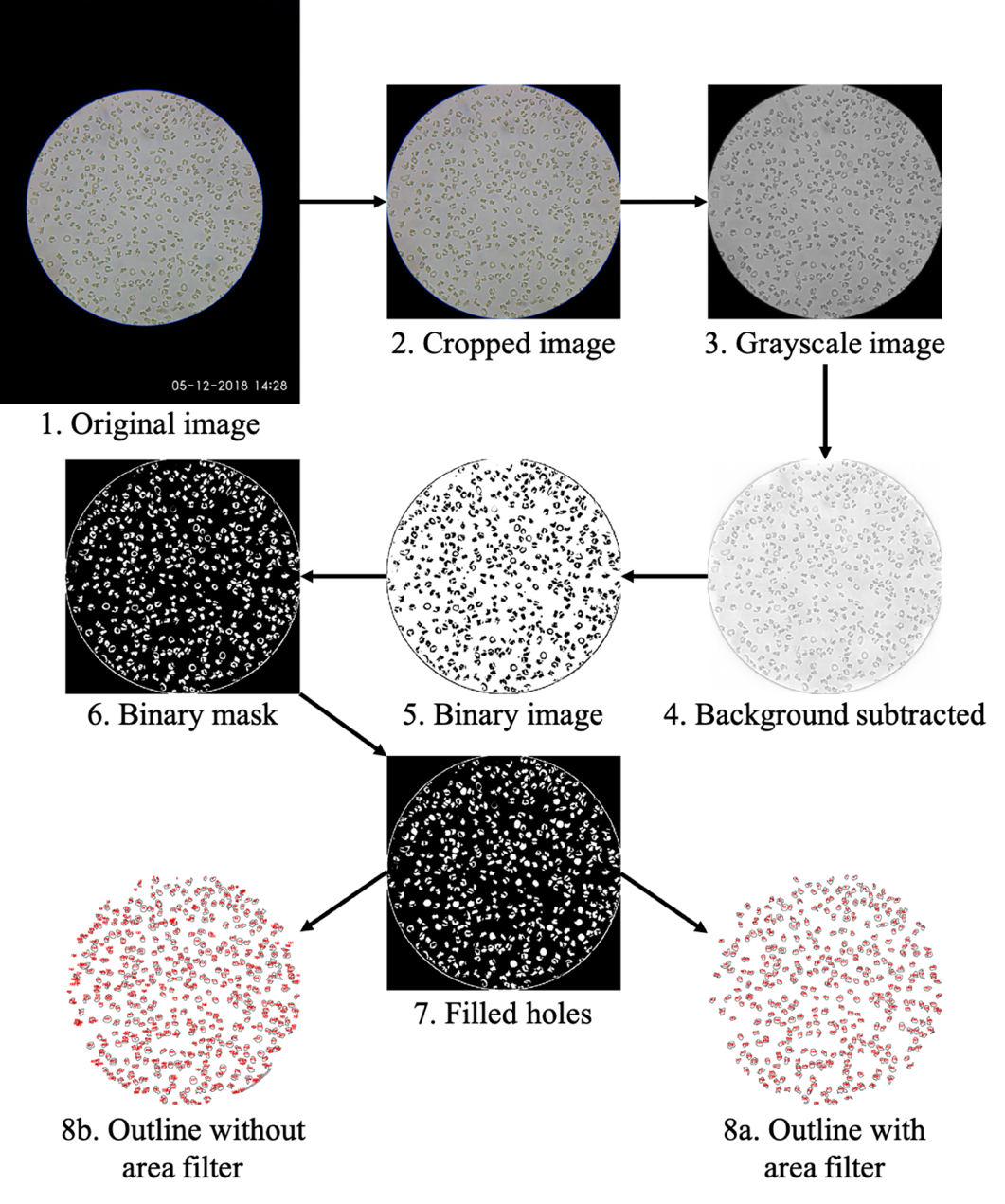


***Figure S4****. Steps involved in image analysis are shown. The original image (1) is opened in ImageJ and the area of interest is cropped (2). Then image is converted into an 8-bit grayscale image (3) and its background is subtracted to bring the RBCs in the foreground (4). The image is then binarized (5) and converted into a binary mask (6) to get the RBCs as white particles. The holes in the particles are filled to fill the center of the unfocused part of biconcave RBCs (7). Then the RBCs are analyzed to get their solidity, roundness values and outlines. 8a shows the outlines of the RBCs when an area filter is applied to fit the RBC sizes, while 8b shows the outlines without an area filter.*

**7. Choosing ‘roundness’ as the shape descriptor**


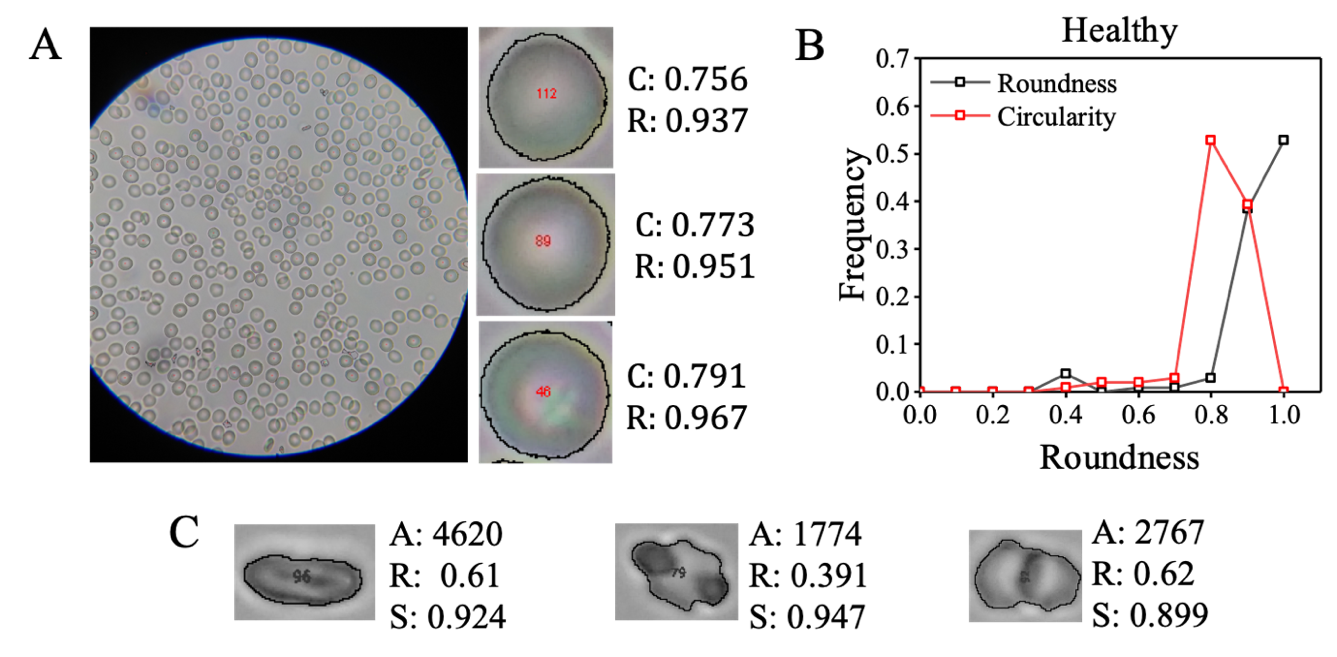


***Figure S5****. Roundness as a shape descriptor for quantifying RBC shapes in healthy and sickle blood. (A) Image of a healthy blood sample shows biconcave RBCs. Circularity and roundness values of three RBCs from this image are also shown. (B) The roundness distribution plot of RBCs in this image peaks at 0.9-1.0, while circularity distribution plot peaks at 0.8. (C) Images of selected RBCs from a healthy sample (ID: N03) showing RBCs lying sideways (left panel), crenated RBCs (middle panel) or overlapping RBCs (right panel). A, R, and S indicate area, roundness and solidity respectively.*

**Figure S5A** shows the image of a healthy blood sample. Three individual RBCs, numbered as 112, 89 and 46 in this image, are shown separately with their respective ‘circularity’ and ‘roundness’ values. As these three RBCs appear almost circular, we expect both roundness and circularity values to be very close to 1. While all the roundness values are higher than 0.9, the circularity values are only around 0.7 (**Figure S5B**). This is because ‘circularity’ uses the value of the perimeter of an object, which is very sensitive to the accurate detection of the boundary pixels. It works well with very high-resolution images acquired by expensive microscopes. As the formula for roundness does not rely upon accurate detection of the boundary pixels, it can work for images acquired by both high-end and inexpensive microscopes. We chose to work with roundness to avoid artefacts while detecting the outlines of sickle cells and wrongly classifying their shapes. The roundness distributions of healthy samples have tails at values of R<0.8. As shown in **figure S5C**, this is due to the presence of certain artifacts such as (a) biconcave RBCs lying sideways (left panel), (b) crenated RBCs (middle panel), and (c) two or more overlapping RBCs (right panel). However, as our validation data shows, our workflow is robust even with the presence of these artefacts.


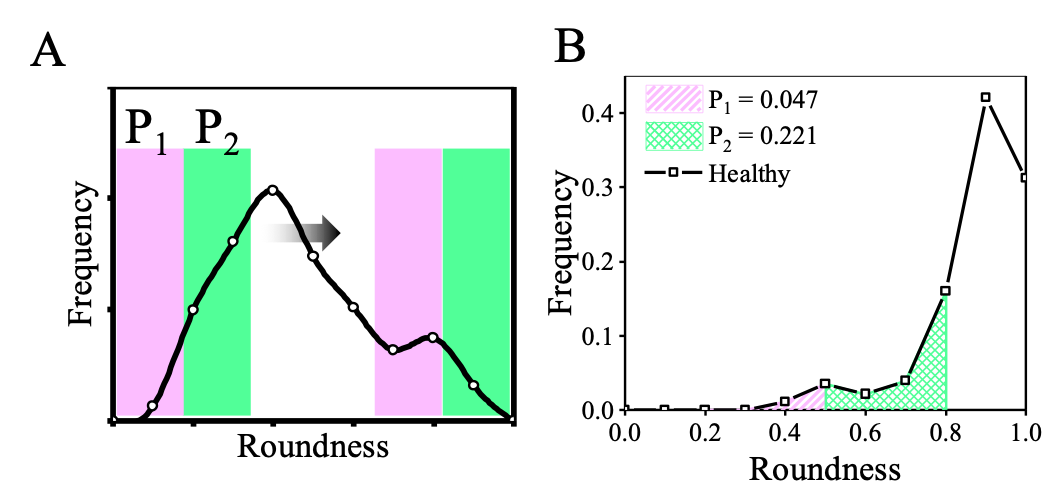
**8.** **Defining two shape descriptors** $\boldsymbol{P}_{\boldsymbol{1}}$ **and** $\boldsymbol{P}_{\boldsymbol{2}}$

***Figure S6****. Development of classifiers from our training dataset. (A) In this schematic diagram, we explain how we define the two secondary shape parameters* $P_{1}$ *and* $P_{2}$*.* $P_{1}$ *is the area under the roundness curve indicated in violet and* $P_{2}$ *is the area under the roundness curve indicated in green. In this way we transform the information contained in the shape of each roundness distribution to a single point given by the coordinates* $P_{1}$ *and* $P_{2}$ *in the* $P_{1}-$ $P_{2}$ *parameter space. (B) A typical illustration of* $P_{1}$ *and* $P_{2}$ *from the roundness plot of a healthy blood sample.*

Since comparing two numbers is simpler than comparing the shapes of two irregular-shaped curves, our objective is to transform the information contained in each roundness distribution plot into a point given by two coordinates ($P_{1}$, $P_{2}$). The position of the primary peak of the roundness distribution plot or the area under the curve are potential choices for such a number. We find that the SCD and SCT distributions for $C_{high}$ overlap, while the SCT and healthy distributions for $C_{low}$ overlap. Consequently, using the peak positions to distinguish between these samples is not feasible. All our roundness plots are normalized such that the area under each curve is unity. Hence, we focus on two specific areas under a roundness curve instead, shown by the violet and green rectangles in **Figure S6A**, and call them $P_{1}$ and $P_{2}$. The information about the RBC shapes in any image is now represented by the coordinates ($P_{1}$, $P_{2}$) of a point in this new parameter space (i.e., in a plot of $P_{2}$vs. $P_{1}$). **Figure S6B** shows one set of $P_{1}$ and $P_{2}$ values obtained from the roundness curve of a healthy blood sample.

To determine the most appropriate choice of $P_{1}$ and $P_{2}$, we translate two contiguous sections of equal width (violet and green) by increments of 0.1 over the entire range of roundness values, while also varying their widths from 0.2 to 0.5. It is obvious that the values of $P_{1}$ and $P_{2}$ will lie between 0 and 1. Now, there are 16 possible combinations of $P_{1}$ and $P_{2}$ covering the entire range of roundness values from 0 to 1, as shown in **Table T3**, and we need to identify the most optimal condition.

***Table T3****: 16 possible combinations of* $P_{1}$ *and* $P_{2}$*.*

**
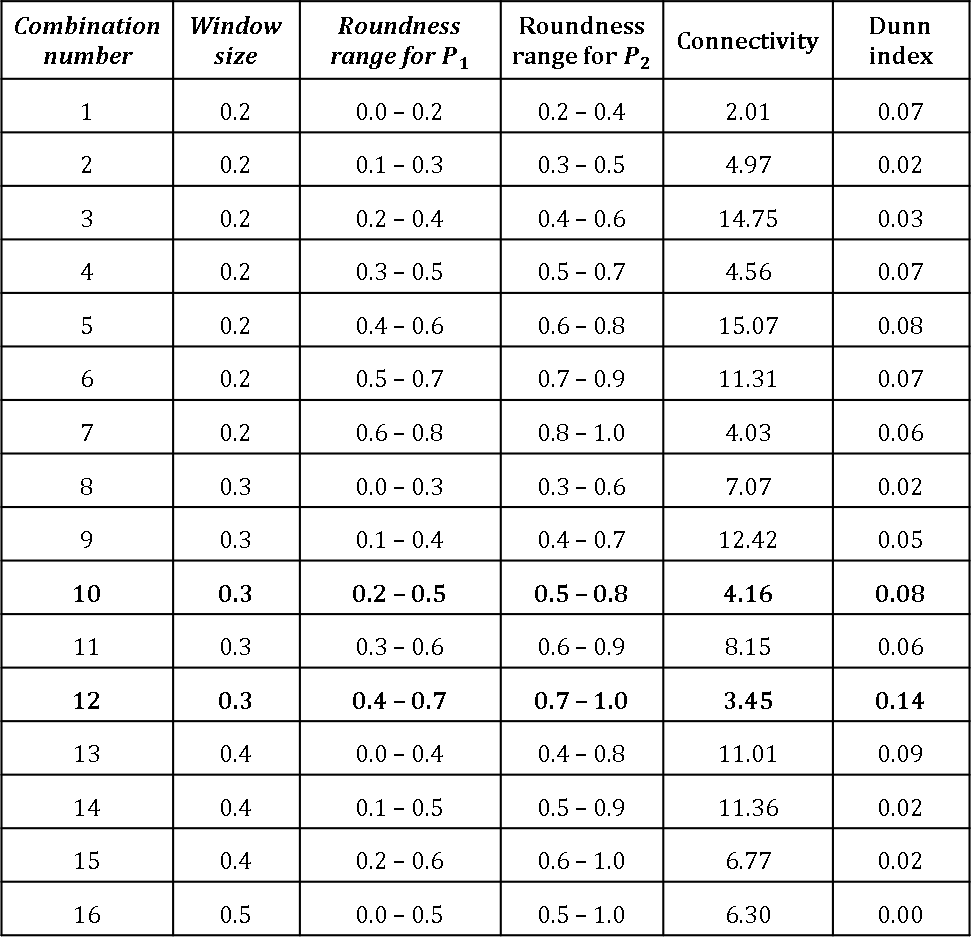
**

**9. Choosing the most optimal values of** $\boldsymbol{P}_{\boldsymbol{1}}$ **and** $\boldsymbol{P}_{\boldsymbol{2}}$

We plot the training dataset of 164 known samples (H = 49, D = 23 and T = 92) in the $P_{1}-$ $P_{2}$ space. Each image is now indicated by a point in this space. The training dataset of 164 samples is well separated into two clusters, one corresponding to healthy blood and the other corresponding to sickle blood (both SDC and SCT). We calculate the connectivity and Dunn index for each of these 16 combinations. Connectivity indicates how strongly two clusters are connected, while Dunn index indicates intra-cluster compactness and inter-cluster separation. **Table T3** lists the connectivity and Dunn index values of clusters corresponding to all 16 combinations of $P_{1}$ and $P_{2}$. We plot Dunn index vs. connectivity (**Figure S7)** to identify the most robust combination that has low connectivity and high Dunn index. These points will lie in the top right quadrant of the plot.

***Figure S7.*** *A plot of Dunn index vs. connectivity for all 16 possible clusters.*

As shown in **Figure S8,** both combination #10 and combination #12 appear to be optimal. Combination #10 spans roundness values from 0.2 to 0.8, with $P_{1}$ranging from 0.2 to 0.5 and $P_{2}$ ranging from 0.5 to 0.8. Combination #12 spans roundness values from 0.4 to 0.1 with $P_{1}$ ranging from 0.4 to 0.7 and $P_{2}$ ranging from 0.7 to 1.0. The top panels indicate that healthy (black square) and sickle (red circle and blue triangle) clusters are well separated in both these combinations. As indicated in the lower panels, combination #10 includes information about sickle RBCs (0.2 < R < 0.4) and excludes unsickled RBCs (R > 0.8). As shown in **Figure S8**, Combination #12 lacks information about sickle RBCs (R < 0.4) but includes unsickled RBCs (R > 0.8). Consequently, we proceed with combination #10 for further analysis.

*
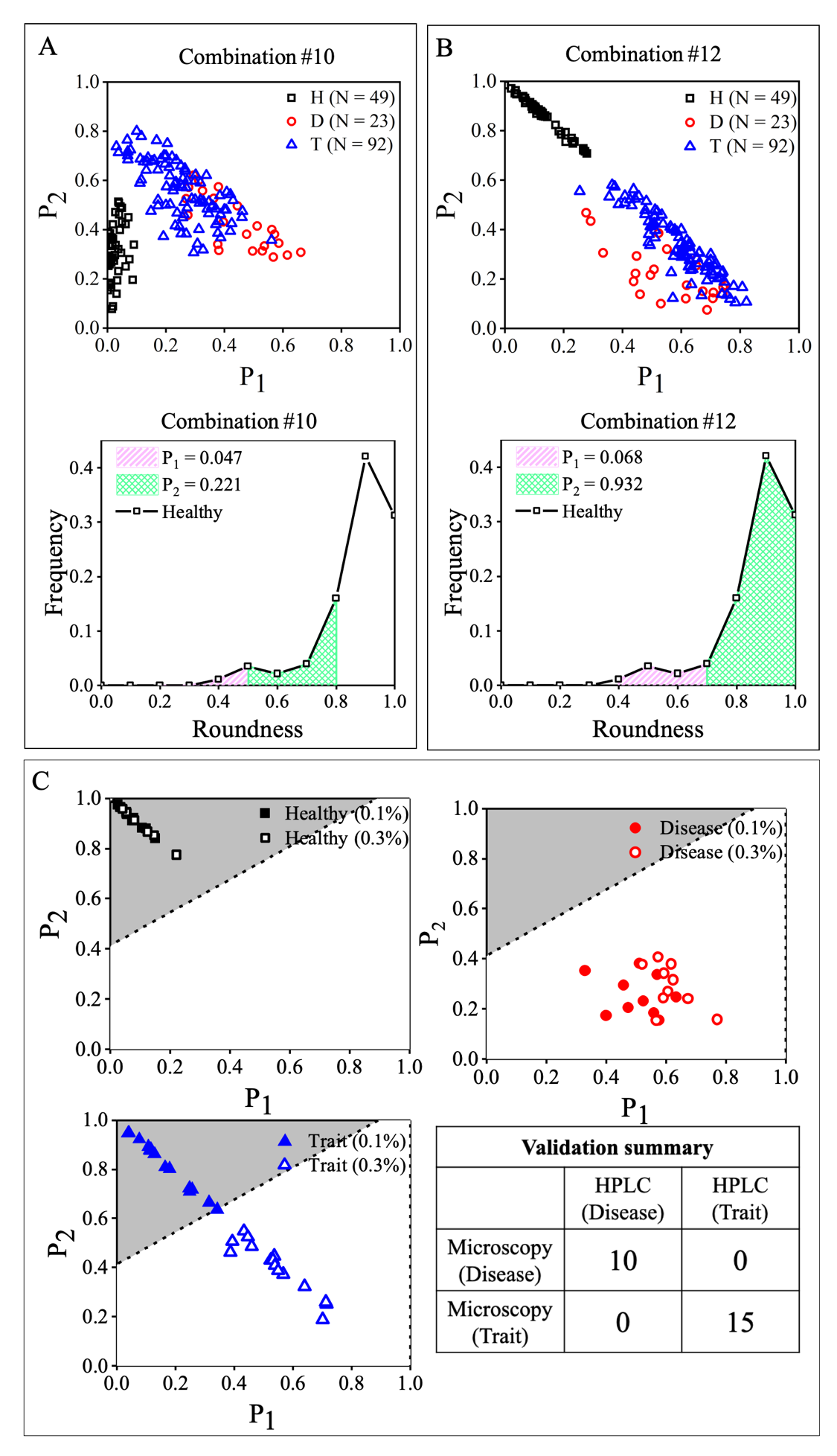
*

***Figure S8.*** *(A, B) Comparison of combinations #10 and #12.
(C) Validation data using classifier combination #12.*

**
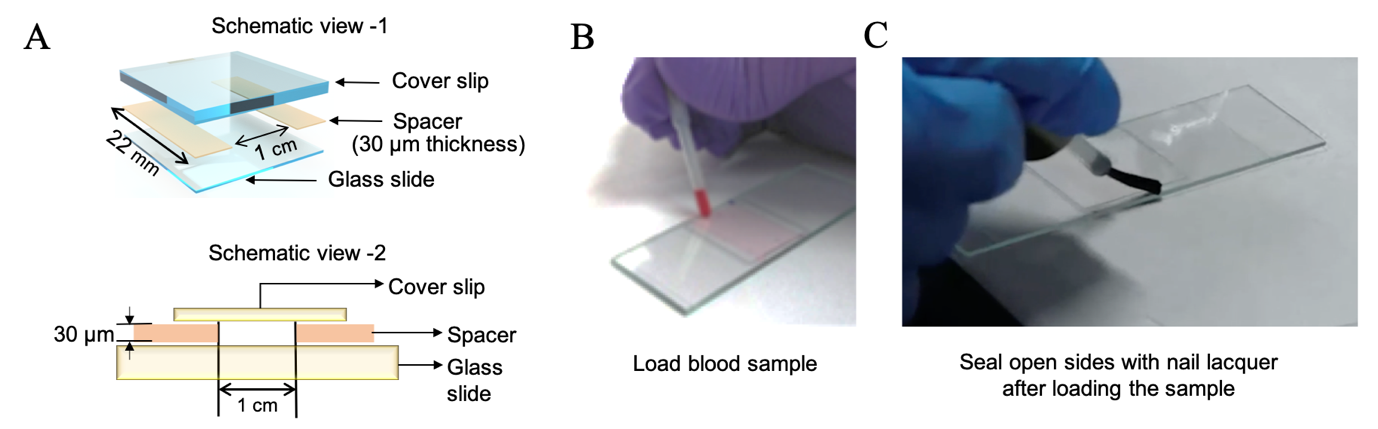
10. Imaging microchamber**

***Figure S9.*** *(A) Schematic diagram showing the structure and dimensions of the imaging microchamber. It is 22 mm X 10 mm X 30 µm in dimension. (B) Two opposite sides of the imaging chamber are open to allow loading of blood as shown. (C) Once blood is loaded, the two open sides are closed using a quick drying nail lacquer to avoid the blood sample from coming in contact with atmospheric oxygen.*

**
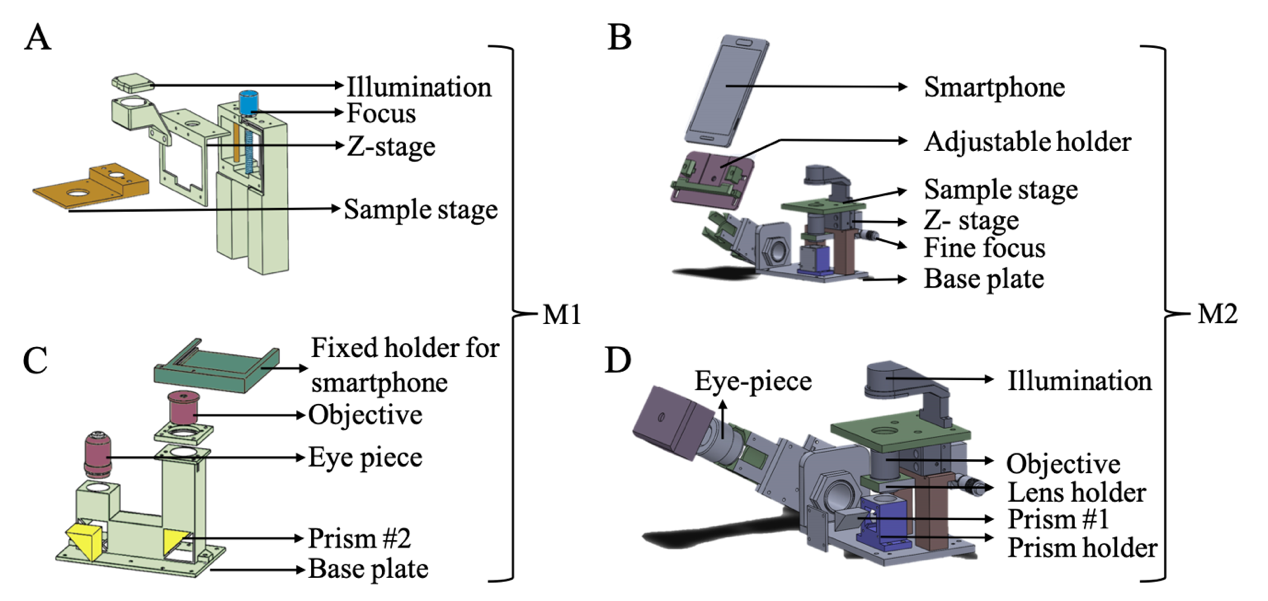
11. Optical and mechanical designs of the portable microscope**

***Figure S10****. CAD diagrams showing the optical (A, C) and mechanical (B, D) designs of the smartphone microscope models with fixed phone holder (M1) and adjustable phone holder (M2) respectively.*

**Figures S10A** and **S10B** show the exploded views of the mechanical parts of the smartphone microscope models with fixed phone holder M1 and adjustable phone holder M2 respectively. The microfluidic chip is placed on the sample stage. The sample stage, and not the objective, is moved to focus the image on the camera. The focusing arrangement and the base plate are made of aluminium to make the microscope stable. The remaining parts and the outer casing of the microscope are 3D-printed in polylactic acid (PLA). **Figures S10C** and **S10D** show the exploded views of the optical assembly inside M1 and M2 respectively. In the case of M2, a rotating arm ensures that (i) the incident surface of the second prism is parallel to the emitting surface of the first prism during rotation, and (ii) the emitting surface of the second prism is parallel to the eyepiece. The smartphone holder in both models is customizable to specific phone models and the phone is removed from the holder when not in use. We use Xiaomi Mi3 or Samsung A10 phones to capture images with a minimum resolution of 2368 pixels x 4208 pixels. These phone models are chosen to balance performance, cost and availability in developing countries.

**12. Captions of movies uploaded as supporting information**

**Movie M1:** Sickling video of a disease sample treated with 0.1% sodium metabisulphite. The 30 min video has been sped up 29.5 times. The sample ID is D188.

**Movie M2**: Sickling video of a disease sample treated with 0.3% sodium metabisulphite. The 30 min video has been sped up 29.8 times. The sample ID is D188.

**Movie M3**: Sickling video of a trait sample treated with 0.1% sodium metabisulphite. The 30 min video has been sped up 29.5 times. The sample ID is T187.

**Movie M4**: Sickling video of a trait sample treated with 0.3% sodium metabisulphite. The 30 min video has been sped up 29.5 times. The sample ID is T187.
